# Method of meal delivery influences estimates of postprandial metabolism and alkaline tide in a benthic fish

**DOI:** 10.1111/jfb.70678

**Published:** 2026-09-30

**Authors:** Junlin Chen, Barrett W. Wolfe, Harriet R. Goodrich

**Affiliations:** ^1^ Institute for Marine and Antarctic Studies (IMAS), University of Tasmania Hobart Tasmania Australia

**Keywords:** acid–base regulation, bicarbonate, gastrointestinal physiology, gavage, *Platycephalus bassensis*, specific dynamic action

## Abstract

Intragastric gavage is widely used in studies of digestive physiology to deliver standardised meals, yet it remains unclear whether it elicits postprandial metabolic and acid–base responses comparable to voluntary feeding. Here, we tested whether the method of meal delivery affects the magnitude and timing of the postprandial rise in metabolic rate known as specific dynamic action (SDA) and the associated alkaline tide (transient rise in blood pH and bicarbonate following feeding) in sand flathead (*Platycephalus bassensis*) using a standardised 1.5% body‐weight ratio either as intact pellets (voluntary feeding) or as a homogenised slurry via gavage, alongside fasted and sham‐gavage controls. SDA magnitude was ~3.6‐fold greater and duration was ~37 h longer in voluntarily fed fish. Removing the post‐handling period reduced this difference, and the early post‐entry period accounted for a greater proportion of metabolic expenditure in voluntarily fed fish, suggesting apparent differences partly reflect procedural effects of feeding method, including the mandated use of anaesthesia during the gavage procedure. Voluntarily fed fish showed a clear alkaline tide, to our knowledge the first report in this species, whereas gavage‐fed fish showed no tide at 6 h, with lower blood pH and a delayed bicarbonate rise. Gut content distribution at 6, 12 and 24 h post feeding indicated reduced temporal separation between gastric acid secretion and intestinal neutralisation in gavage‐fed fish. These results show that method choice can modify digestive physiology in ways that should be considered when interpreting and comparing studies of postprandial responses in fish.

## 1 INTRODUCTION

A major challenge in studies of feeding physiology is the need to obtain measurements from animals in a standardised postprandial state. Although feeding may appear straightforward in principle, controlling variables such as meal size, meal composition, feeding time and the interval since feeding can be difficult, particularly when voluntary food intake is variable among individuals or species, or is suppressed under experimental conditions. Consequently, the method of meal delivery is often adapted to suit the species being studied and the logistical constraints of the experimental design. In fish, meals are typically delivered either by voluntary feeding on intact food items or by intragastric gavage of a homogenised slurry. Following a review of studies in fish gastrointestinal physiology, we estimate that approximately 32% of experiments use gavage rather than voluntary ingestion (Tables S1 and S2). Despite this widespread use, little attention has been given to whether the two approaches elicit comparable physiological responses.

Intragastric gavage, the delivery of a homogenised meal directly into the stomach via an orogastric tube under light anaesthesia, is routinely used in studies of fish postprandial metabolism, nutritional physiology and pharmacology (Belal, 2005; Collymore et al., 2013; Cooper & Wilson, 2008; Jobling, 1994; Secor, 2009). Its primary advantage lies in the precise and repeatable control it provides over ration size, meal timing and dietary composition, which is particularly valuable when comparing physiological responses among diets, life stages, or species, or when studying animals that feed unreliably under laboratory conditions (Belal, 2005; Collymore et al., 2013; Cooper & Wilson, 2008). However, the physical and behavioural context of feeding also strongly influences digestive physiology, and gavage departs from voluntary feeding in several important respects. Gavage bypasses prey capture and ingestion behaviours, removes much of the sensory and cephalic stimulation that normally precedes feeding (Cooper & Wilson, 2008), must be performed under anaesthesia (Collymore et al., 2013) and delivers food to the stomach in a highly hydrated and mechanically processed form (Cooper & Wilson, 2008). Anaesthetic agents are themselves known to influence metabolic rate, cardiovascular function and acid–base status, representing an additional source of between‐study variation that is rarely acknowledged when comparing gavage and voluntary‐feeding paradigms (Iwama et al., 1989; Zahl et al., 2012; Herrera‐Castillo et al., 2024). Converting intact pellets into a slurry substantially alters physical characteristics such as particle size, viscosity and surface area‐to‐volume ratio, all of which influence gastric retention time and emptying kinetics (Bromley, 1994; Garber, 1983; Grove et al., 1978; Jobling, 1987). These processes are closely linked to the timing and magnitude of postprandial physiological responses, including specific dynamic action (SDA) and the postprandial alkaline tide.

SDA refers to the transient elevation in metabolic rate that follows feeding and reflects the energetic costs associated with digestion, absorption, nutrient processing and assimilation (Jobling, 1981; McCue, 2006; Secor, 2003, 2009). Among fishes, SDA has been quantified across a wide range of teleosts and elasmobranchs (Beamish, 1974; Chabot et al., 2016; Goodrich et al., 2024; Jobling & Davies, 1980) and is known to be strongly influenced by the physical characteristics of a meal and environmental conditions such as temperature (Jutfelt et al., 2021; Secor, 2009). Food particle size, texture, hydration state, dietary buffering and degree of prior processing all alter digestive kinetics and nutrient assimilation rates, thereby shaping the timing, magnitude and duration of total postprandial metabolic rate (Bromley, 1994; Garber, 1983; Goodrich, Berry, et al., 2022; Jobling, 1987; Tandler & Beamish, 1980).

In some gastric species, SDA has been shown to be accompanied by a coordinated acid–base disturbance known as the postprandial alkaline tide. During gastric digestion, hydrochloric acid is secreted into the stomach lumen by oxyntopeptic cells, while an equimolar quantity of bicarbonate (HCO_3_^−^) is released into the bloodstream via basolateral Cl^−^/HCO_3_^−^ exchange (Boutilier et al., 1984; Niv & Fraser, 2002). The resulting transient increase in systemic blood pH and plasma bicarbonate concentration is subsequently compensated for, primarily at the gills, through branchial ion‐transport processes involving H^+^‐ATPases, Na^+^/H^+^ exchangers and Cl^−^/HCO_3_^−^ exchangers (Claiborne et al., 2002; Evans et al., 2005; Parks et al., 2007; Perry & Gilmour, 2006; Tresguerres et al., 2007; Wood, 2019). The magnitude and temporal expression of the alkaline tide are therefore closely linked to gastric processing dynamics, and in particular to retention time in the stomach. Intact food particles, or food with a high buffering capacity, are expected to be retained in the stomach for prolonged trituration and acid digestion before gradual evacuation into the intestine (Goodrich, Berry, et al., 2022; Goodrich, Wilson, et al., 2022). Extended retention time allows the alkaline tide to develop in the systemic circulation before intestinal bicarbonate utilisation increases substantially (Taylor & Grosell, 2009). In contrast, homogenised liquid meals, such as those delivered by gavage, are expected to empty more rapidly from the stomach, potentially compressing the temporal separation between gastric acid secretion and intestinal neutralisation (Cooper & Wilson, 2008; Garber, 1983). Rapid gastric evacuation may also alter intragastric pH regulation and feedback control of acid secretion through effects on luminal buffering and gastrin signalling (Dockray et al., 2001; Niv & Fraser, 2002; Yúfera & Darías, 2007). Despite these mechanistic differences, the consequences of gavage feeding for postprandial acid–base balance and metabolism remain poorly resolved, and comparisons among studies using different feeding methods may be confounded in ways that are currently underappreciated (Belal, 2005; Cooper & Wilson, 2008).

To address this knowledge gap, this study used wild‐caught sand flathead (*Platycephalus bassensis* Cuvier, 1829), a demersal, site‐attached ambush predator that strikes at passing prey from partial burial in the sediment. Like other platycephalids it is a generalist carnivore taking teleosts and benthic crustaceans (Barnes et al., 2011; Goodrich et al., 2026), although its gastrointestinal physiology remains poorly characterised, with the available work limited to early descriptions of gastric motility and enteric innervation (Anderson & Campbell, 1988; Grove & Campbell, 1979). In this species, we quantified total SDA following either voluntary ingestion of intact pellets or intragastric gavage of a homogenised slurry, with both treatments receiving a standardised ration equivalent to 1.5% of body mass. We then characterised the temporal dynamics of postprandial blood pH, PCO_2_ and [HCO_3_^−^] via serial blood sampling of fish when fasted, and 6, 12, 24 and 48 h post‐feeding, to determine whether the method of food delivery alters the timing, magnitude or direction of the postprandial alkaline tide. We hypothesised that (i) voluntary feeding would produce a greater SDA magnitude and longer SDA duration than intragastric gavage and (ii) voluntarily fed fish would exhibit an earlier and more pronounced rise in blood pH and blood bicarbonate compared to gavage‐fed fish and would require a longer time to return to pre‐feeding acid–base status (Cooper & Wilson, 2008; Goodrich, Berry, et al., 2022; Goodrich, Wilson, et al., 2022; Moffatt et al., 2022; Taylor & Grosell, 2009).

## 2 MATERIALS AND METHODS

### 2.1 Ethics statement

All research was carried out in accordance with the Australian Code for the Care and Use of Animals for Scientific Purposes and approved by the University of Tasmania Animal Ethics Committee (Approval No. A40081). Collection of wild fish was authorised under the Living Marine Resources Management Act 1995, section 12 Scientific Research Permit No. 25075. The holding and maintenance of animals in captivity for research purposes were authorised under the Living Marine Resources Management Act 1995, section 12 Scientific Research Permit No. 25181. Fish were euthanised at the end of all experimental procedures by immersion in an overdose of AQUI‐S® (50 mg L^−1^).

### 2.2 Literature survey

To estimate the relative use of voluntary feeding versus intragastric gavage in fish postprandial physiology research, we conducted a representative survey of primary research articles published between 1972 and 2025. Studies were identified through Google Scholar searches using combinations of the terms ‘fish’, ‘specific dynamic action’, ‘SDA’, ‘postprandial metabolism’, ‘digestion’, ‘gavage’, ‘force‐fed’ and ‘voluntary feeding’, supplemented by manual searches of reference lists from key studies and reviews (e.g., Chabot et al., 2016; Secor, 2009) to ensure historical coverage. Studies were included if they: (i) experimentally fed fish, (ii) measured a postprandial metabolic, acid–base or digestive variable, and (iii) explicitly stated the method of meal delivery. We excluded review articles, larval fish studies and papers lacking clear feeding methodologies. Based on the methods described in each study, meal delivery was classified as voluntary feeding or intragastric gavage. While not an exhaustive systematic review, this compilation provides a cross‐section of prevalent feeding methodologies in the field. The results of the survey are collated by taxa and method in Table  and full list of studies and classifications is provided in Table S2.

### 2.3 Capture, transport and animal husbandry

Sand flathead (*Platycephalus bassensis*; *n* = 69, 141.63 ± 5.84 g, 27.80 ± 0.43 cm total length) were captured via baited hooks from the Derwent Estuary (Tasmania, Australia) and transported to the Temperate Marine Laboratory at IMAS Taroona in aerated containers filled with natural seawater. Upon capture, fish were removed using a de‐hooking tool and visually assessed, with only individuals in good condition (no visible abrasions, deformities or hook injury) retained for experimental use. Fish were immediately transferred to aerated holding tubs (∼70 L) supplied with ambient seawater (15–17°C) at a density not exceeding ~25 kg m^−3^ (approximately 6–8 fish per tub). Dissolved oxygen was maintained above 90% air saturation using portable aeration, and water temperature was continuously monitored and maintained within ~1°C of ambient sea temperature. Seawater within holding tubs was partially exchanged at regular intervals during fishing to maintain water quality. Upon arrival at the laboratory, fish were gradually acclimated to the recirculating aquaculture system (RAS) by incremental replacement of transport water with system water over ~30 min.

Fish were distributed across 16 circular 300 L tanks within a 10,000 L recirculating aquaculture system arranged in a two‐tier rack configuration. The system was supplied with seawater from the Derwent Estuary, which underwent mechanical filtration (50 μm) and UV sterilisation prior to entering temperature‐controlled sump reservoirs. Water was circulated through the system via a central pump, delivered to tanks through a manifold and returned via standpipe overflows for continuous recirculation. To facilitate natural behaviours, all tanks were fitted with a sand substrate that allowed fish to bury. System water temperature was maintained at 15 ± 0.3°C (RAS temperature settings) to match sea surface temperature at capture. Temperature was maintained throughout the study using integrated heater–chiller units (Rheem Australia Pty Ltd., Australia) in combination with inline compact heaters (Optima, Electro Engineering Ltd., UK). Throughout the trial, water parameters (presented as mean ± SEM unless otherwise stated) were maintained at pH 8.11 ± 0.03, NH_3_ 0.23 ± 0.01 mg L^−1^, NO_2_ 0 ± 0 mg L^−1^, NO_3_^−^ 0 ± 0 mg L^−1^ and salinity 37.19 ± 0.36. Fish were maintained under a controlled photoperiod using programmable LED lighting (Fluval Smart LED, Fluval, Italy), set to a 16 h light: 8 h dark cycle (lights on 0600 h, lights off 2200 h), with gradual 1 h transitions at dawn (0600–0700 h) during which light intensity ramped up progressively, and a two‐stage dusk transition in which all light channels were gradually dimmed in synchrony from 1800 h to 1900 h, followed by a constant low‐intensity blue light from 1900 h to 2200 h, to simulate natural twilight and minimise behavioural disturbance. During acclimation, fish were offered commercial sinking pellets daily at 08:00 for 15 min. Pellets were distributed close to each fish and offered in excess until no further feeding was observed, providing an operational measure of apparent satiation. Routine acclimation intake was not weighed and therefore cannot be expressed retrospectively as a percentage of body mass. Each fish was acclimated for at least 7 days; because experiments were conducted in batches, some fish remained longer. Fish generally began accepting commercial sinking pellets within 3 days.

### 2.4 Feeding treatments and procedure

Following acclimation, 20 fish entered the SDA‐respirometry experiment (five batches of four fish) and 47 entered the terminal blood‐sampling experiment. The four treatments were voluntary feeding, gavage feeding, fasting and procedural controls (sham gavage). Prior to experiments, fish were lightly anaesthetised in situ using Aqui‐S® (15 mg L^−1^) to allow body mass measurement and transfer to individual 300 L tanks connected to the recirculating aquaculture system described above. For the feeding treatment, all fish were fed the same commercial sinking pellet diet with the ration standardised to 1.5% body mass, delivered as a single meal. A 1.5% value was determined from our own observations of the amount that fish would reliably consume voluntarily within a single feeding session, and was therefore set below apparent satiation to make complete consumption within 15 min feasible and to allow exact ration matching between treatments.

Voluntarily fed fish were offered the ration as intact pellets between 0800 h and 1100 h, the pre‐weighed ration was offered one pellet at a time over a maximum of 15 min through a small opening in the tank cover while the observer remained outside the fish's direct line of sight. Consumption of every pellet was confirmed visually. If a fish did not consume the complete ration, that trial was terminated and the fish was fasted for a further 48 h and subsequently retested. For gavage treatments, the same ration was provided as a slurry prepared immediately prior to use by grinding pellets to a fine powder using a conical burr grinder (Sunbeam EM0440 GrindFresh, grind setting 5) and mixing with RAS seawater at a ratio of 1:1.3 (feed to water, w:v). The slurry was mixed thoroughly to ensure complete hydration and loaded into a 10 mL bird crop syringe (MedCart, Australia). Gavage was performed under benzocaine anaesthesia using a two‐stage protocol. Fish were first sedated in situ using 35 mg L^−1^ benzocaine until loss of equilibrium and reduced response to handling, then transferred to a secondary anaesthetic bath containing 75 mg L^−1^ benzocaine to achieve surgical anaesthesia following best practice from Davison et al. (2023). Fish were removed from the bath and a curved stainless steel gavage tube (10 G, ~130 mm total length, ~5.5 mm ball tip) attached to the syringe was inserted orally and advanced to the level of the stomach, approximately in line with the base of the pectoral fins. The slurry was delivered at a controlled rate of ~0.5 mL s^−1^. Following dispensing of the full 1.5% ration, the tube was withdrawn immediately and the fish was either returned to recovery tanks supplied with aerated system water for subsequent blood sampling or transferred into the respirometry system for measurement of SDA. If any regurgitation or meal loss was observed, the trial was terminated and excluded, and the fish was returned to its holding tank for 48 h of fasting before retesting. Procedural control fish underwent the same anaesthesia and intubation procedure, including full tube insertion and equivalent handling time, but received no slurry.

### 2.5 Respirometry

Following completion of treatment procedures, fish were immediately transferred to individual respirometry chambers. Voluntary‐fed and fasted fish were transferred by hand‐netting without anaesthesia, whereas gavage‐fed and sham‐gavage fish were transferred while under benzocaine anaesthesia as associated with the procedure described above.

#### 2.5.1 Respirometry system

SDA was quantified using automated intermittent‐flow respirometry to measure rates of oxygen consumption (ṀO_2_), following the system and protocol described in Goodrich et al. (2026). The system consisted of four sealed acrylic respirometry chambers (3.15 L; Eagle Plastics) submerged within a 300 L recirculating seawater bath (salinity 37) maintained at 15 ± 0.5°C using a TECO TK500 chiller (TECO Refrigeration Technologies, Italy). The water bath was housed in a temperature‐controlled room maintained at 13 ± 0.5°C in order to limit environmental heat transfer into the bath and slow warming should chilling fail. Each chamber was fitted with a Loligo Systems respirometry lid and equipped with two independent water circuits. An internal recirculation loop (Eheim Universal 1046 pump) continuously mixed water and passed it through a flow‐through cell housing a fibre‐optic oxygen sensor (SPFIB‐BARE optode; PyroScience GmbH, Germany). An external flush circuit (Tetra WP300 pump) intermittently replaced chamber water with fully oxygenated bath water, with flow regulated via an in‐line valve and directed through a one‐way valve to prevent backflow during sealed measurement phases. Dissolved oxygen and temperature were recorded continuously using a 4‐channel FireSting oxygen meter and temperature probe (PyroScience GmbH, Germany), with data logged via Aqua OxyLog and system cycling controlled by AquaResp 3 software (Svendsen et al., 2019) in combination with a USB‐controlled Cleware switch.

#### 2.5.2 Respirometry protocol and data processing

Respirometry operated on a repeating flush–wait–measure cycle controlled by AquaResp 3 (Svendsen et al., 2016, 2019). Each cycle lasted 25 min and consisted of: (1) a flush phase (240 s), during which chamber water was replenished to ≥90% air saturation; (2) a wait phase (60 s), allowing dissolved oxygen to homogenise before measurement; and (3) a sealed measurement phase (1200 s), during which the decline in dissolved oxygen was recorded. Rates of oxygen consumption (ṀO_2_) were then calculated from the slope of oxygen decline during the measurement phase. To account for microbial oxygen consumption within the chambers and tubing, background respiration was measured in fish‐free chambers immediately following each experimental trial (‘blanks’) and subtracted from fish ṀO2 as follows. Data points within an oxygen saturation range of 90%–120% were retained during data processing. In cases where calculated background respiration was negative (i.e., an apparent increase in PO_2_ over time), background ṀO_2_ was set to zero. This reflects a known physical artefact in sealed respirometry systems, where minor thermal input from recirculating pumps can elevate temperature within the chamber, reducing oxygen solubility and increasing measured PO_2_ despite no change in dissolved oxygen content (Henry's law). As background respiration was negligible or negative in all trials (<1% of absolute ṀO_2_), no corrections were applied. Following the completion of each trial and background respiration blank, the respirometry system was cleaned by physical scrubbing to remove mucus and biofilms, followed by immersion in a 0.5 mL L^−1^ sodium hypochlorite solution for ≥30 min and thorough rinsing with fresh water.

All raw respirometry data were imported into R (v4.5.2; R Core Team, 2025) and processed using the *flatheadresp* package (Wolfe, 2026). For each measurement cycle, dissolved oxygen concentration recorded during the sealed measurement phase was extracted and regressed against time to estimate the rate of oxygen decline (ΔPO_2_ Δt^−1^). These slopes were converted to mass‐specific oxygen consumption rates (ṀO_2_) using oxygen solubility at the recorded temperature, and the volume of water calculated from chamber volume minus individual fish volume estimated from fish mass and an empirical density for the species of 1.1 kg L^−1^. Data quality was assessed for each cycle using the coefficient of determination (*R* ^2^) of the linear regression, and cycles with *R* ^2^ < 0.90 were excluded as exhibiting non‐linear or disturbed oxygen decline, for example due to behavioural disturbance or minor system artefacts.

To standardise metabolic rates across individuals of differing body mass and to enable valid inter‐individual comparisons, mass‐specific ṀO_2_ values were adjusted for allometric scaling to standardise metabolic rates among fish of different body masses. An a priori mass‐scaling exponent of 0.79 was used, corresponding to the mean exponent reported across fishes by Clarke and Johnston (1999):

$${\mathrm{ṀO}}_{2\left(\text{corrected}\right)}={\mathrm{ṀO}}_{2\left(\text{mass}-\text{specific}\right)}\times M^{\left(1-0.79\right)}$$

where M is the wet body mass of the fish in kilograms. Corrected values are expressed in units of mg O_2_ kg^−0^·^79^ h^−1^ (Killen et al., 2010; Norin & Gamperl, 2018).

A two‐pass filtering approach was applied to remove transient spikes in ṀO_2_ associated with spontaneous activity (e.g., startle responses or contact with chamber walls). In the first pass, a sliding window (*k* = 7 cycles) was used to calculate a local median and median absolute deviation (MAD), and observations deviating by >6 × MAD from the local median were flagged as gross outliers. In the second pass, these outliers were temporarily removed, local variance was recalculated and the same threshold was reapplied to identify residual anomalies. This iterative approach prevented inflation of local variance estimates by extreme values and improved detection of secondary deviations. All flagged data points were reviewed against experimental logs, with manual overrides applied where justified by experimental context.

All metabolic parameter calculations were then performed in R using the *fishṀO2* package (Chabot et al., 2016). Standard metabolic rate (SMR) was estimated from data recorded during the final 24 h of each 96‐h trial, when SDA and post‐capture stress effects had subsided. Two estimation approaches were evaluated. The first was the mean of the lowest normal distribution (MLND), derived from a Gaussian mixture model using the *mclust* package. The second was the 20th percentile (q0.2) method. The MLND approach was considered valid only when the coefficient of variation (CV) of the fitted lowest Gaussian component was less than 5.4%. However, only 16.7% of individuals (3 of 18) met this criterion. To ensure consistency across individuals, the q0.2 method was therefore applied uniformly. This approach is widely recommended for datasets with limited sample sizes or elevated metabolic variability (Chabot et al., 2016; Killen et al., 2021). SDA parameters were then quantified using the *calcSDA* function in the *fishṀO2* package. Prior to analysis, ṀO_2_ time series were smoothed using locally weighted regression with parameters τ = 0.5 and λ = 20 to reduce stochastic noise. The end of the SDA response was defined as the point at which the smoothed ṀO_2_ trace returned to within 5% of SMR and remained stable. The following SDA metrics were then calculated. (1) SDA energy represents the integral of ṀO_2_ above SMR and is expressed in mg O_2_ kg^−0^·^79^. (2) SDA peak represents the maximum elevation above SMR during postprandial metabolism. (3) SDA duration represents the time from the onset of elevated ṀO_2_ until return to baseline. (4) Time to peak (TTP) represents the time elapsed from feeding to the moment of peak ṀO_2_. Identical analytical procedures were applied to fasted and procedural control fish to confirm baseline metabolic stability and isolate digestion‐specific responses. To separate metabolic costs associated with digestion from those associated with handling and chamber entry, SDA parameters were calculated using both the full time series following chamber entry, referred to herein as uncorrected, and a post‐acclimation dataset excluding the initial stabilisation period following chamber entry, referred to as corrected. The difference between these estimates provides an index of the metabolic cost of acute handling stress associated with transfer to respirometry chambers.

### 2.6 Postprandial blood acid–base status

Blood samples were collected at targeted post‐treatment time points to quantify postprandial acid–base status and the temporal dynamics of the alkaline tide. Baseline samples were obtained from fasted and procedural control fish (post‐sham gavage), while voluntarily fed and gavage‐fed fish were sampled at 6, 12, 24 and 48 h post‐feeding (*n* = 4–6 per timepoint).

Blood sampling was conducted using an in situ anaesthesia and gill irrigation approach adapted from Davison et al. (2023), designed to minimise respiratory acid–base disturbance associated with air exposure or insufficient gill perfusion (e.g., CO_2_ retention and artificial inflation of blood PCO_2_). Following feeding or procedural treatments, fish were anaesthetised in situ within their holding tanks using benzocaine (35 mg L^−1^), then transferred to a knock‐out bath containing 75 mg L^−1^ of benzocaine until loss of the tail‐pinch reflex. Fish were subsequently positioned in a moulded sponge cradle within a 7 L gill irrigation tray and maintained under a continuous maintenance dose of benzocaine (15 mg L^−1^), delivered via an oral cannula with oxygenated RAS water circulated across the gills using a submersible pump at a rate of 1.1 L min^−1^. The fish was positioned so the anterior portion of the body and gill basket remained submerged, while the caudal peduncle was exposed to allow venipuncture. Whole blood was then collected via caudal venipuncture using a 1 mL syringe fitted with a 25‐gauge needle, pre‐heparinised with lithium heparin. A subsample of whole blood was analysed immediately using a handheld i‐STAT analyser (Abbott Laboratories, USA) with CG4+ cartridges to determine whole‐blood pH, carbon dioxide tension (PCO_2_; mmHg), oxygen tension (PO_2_; mmHg) and lactate. Remaining blood was immediately applied to a portable glucometer (Accu‐Chek®) test strip to measure whole‐blood glucose concentration (mmol L^−1^) as an indicator of acute stress (Mandelman & Skomal, 2009).

As i‐STAT analysers are calibrated for mammalian blood at 37°C, all measurements were corrected to the experimental test temperature (15 ± 3°C) following established temperature‐correction relationships for blood gases (Ashwood et al., 1983; Nunn et al., 1965), which have been validated for use in ectothermic fishes (Damsgaard et al., 2015; Gam et al., 2017; Goodrich, Berry, et al., 2022; Goodrich, Wilson, et al., 2022). Blood pH was therefore corrected as:

$${\mathrm{pH}}_{\mathrm{TC}}={\mathrm{pH}}_{M}-0.015\;\left(T_{M}-37\right)$$

and PCO_2_ as:

$${\mathrm{PCO}}_{\text{2TC}}={\mathrm{PCO}}_{\text{2M}}\times 10^{\left(0.01\text{9ΔT}\right)}$$

where M and TC refer to measured and temperature‐corrected values, respectively, and ΔT represents the difference between analyser and experimental temperature. These corrections account for the temperature dependence of acid–base equilibria, whereby pH increases and PCO_2_ decreases with decreasing temperature.

Blood bicarbonate concentration ([HCO_3_^−^]) was calculated from temperature‐corrected values using the Henderson–Hasselbalch equation:

$$\left[{{\mathrm{HCO}}_{3}}^{-}\right]={\mathrm{\alpha CO}}_{2}\times {\mathrm{PCO}}_{2}\times 10^{\left(\mathrm{pH}-{\mathrm{pK}}^{'}\right)}$$

where the CO_2_ solubility coefficient (αCO_2_) and apparent dissociation constant (pK′) were adjusted for experimental temperature following Boutilier et al. (1984). Temperature‐corrected pH was used as the primary pH variable in all subsequent analyses. Interpretation of calculated [HCO_3_^−^] requires some caution because the precision of i‐STAT PCO_2_ measurements is reduced at the low PCO_2_ values characteristic of water‐breathing fishes (Harter et al., 2014). Any uncertainty in PCO_2_ is propagated through the Henderson–Hasselbalch calculation and may therefore reduce the precision of estimated [HCO_3_^−^] and the ability to detect treatment differences. The direction of the pH and [HCO_3_^−^] responses was nevertheless concordant. We therefore base our principal interpretation of acid–base responses on the directly measured pH signal, with calculated [HCO_3_^−^] used as supporting evidence for the observed patterns.

Following completion of experimental procedures, fish were euthanised by immersion in an overdose of AQUI‐S® (50 mg L^−1^). A ventral incision was then made to expose the gastrointestinal tract for inspection. In fed fish, the degree of digestion and the location of food within the stomach and intestine were recorded, whereas in fasted fish the absence of gastrointestinal contents was confirmed. The gastrointestinal tract of each fish was photographed to provide a visual record of digestive state.

### 2.7 Statistical analysis

All statistical analyses and data visualisation were conducted in R (v4.5.2), using the *dplyr*, *tidyr*, *car*, *emmeans*, *ggplot2*, *fishṀO2*, *effsize* and *TOSTER* packages. Prior to all parametric tests, normality was assessed using the Shapiro–Wilk test and variance homogeneity was evaluated using Levene's test (median‐centred, equivalent to the Brown–Forsythe modification). Where assumptions were met, parametric tests were applied; otherwise, non‐parametric equivalents were used. Specifically, Student's *t*‐tests were used for normally distributed data with equal variances, Welch's *t*‐tests for unequal variances and Wilcoxon rank‐sum tests for non‐normal data. All tests were two‐tailed with α = 0.05, and results are presented as mean ± SEM. SDA metrics, including peak ṀO_2_, total SDA energy (with and without correction for the stress recovery period), the disturbance‐associated component (early post‐entry elevation), SDA duration and TTP, were compared between voluntary‐fed and gavage groups using the pairwise testing rule described above. SMR was compared pairwise among all four treatment groups (fasted, sham gavage, voluntary fed and gavage).

The effects of treatment group and post‐feeding time on blood pH and blood [HCO_3_^−^] were assessed using two‐way ANOVA (Type III sums of squares with sum‐to‐zero contrasts) with Treatment and Time as fixed factors. Because the fasted and procedural control groups were each sampled at only a single time point (0 h and 6 h respectively), the ANOVA was restricted to the voluntary‐fed and gavage groups at the three matched time points (6, 12 and 24 h) to avoid structural imbalance from single‐cell factors. The two gavage fish sampled at 48 h were reported descriptively only and excluded from formal hypothesis tests. ANOVA assumptions were verified using Levene's test on the six cell variances and the Shapiro–Wilk test on the model residuals. Tukey HSD post hoc contrasts were generated using the *emmeans* package both between treatment groups within each time point and within each treatment group across time points; Tukey adjustment was applied within these post hoc families. Because the fasted and procedural control groups could not be incorporated into the two‐way ANOVA, baseline‐referenced contrasts were reported as Hedges' g with 95% confidence intervals to characterise the magnitude of the alkaline tide and acid–base recovery responses; the accompanying p‐values are reported descriptively only and were not entered into the primary hypothesis‐testing framework. Equivalence between the fasted group and the procedural control group was assessed using two one‐sided tests (TOST) applied separately to pH and [HCO_3_^−^]. Equivalence bounds were set at ±0.05 pH units and ± 1.0 mmol L^−1^ [HCO_3_^−^]. The relationship between blood glucose and venous PCO_2_ was assessed using Spearman rank correlation across all fed individuals and time points to evaluate potential contributions of stress related anaerobic metabolism. Respiratory acid–base coupling between blood pH and PCO_2_ was examined in parallel using Spearman correlation, both within fed fish only and across all groups and time points combined. To account for multiple comparisons, *p* values from the SDA family (seven voluntary versus gavage contrasts) and from the SMR family (six pairwise contrasts among the four treatment groups) were each adjusted using the Benjamini–Hochberg false discovery rate procedure. Analyses of potential confounding variables were corrected separately not entered the FDR families. Effect sizes are reported as small‐sample corrected Hedges' g with 95% confidence intervals.

### 2.8 Use of AI

Anthropic Claude (Anthropic, San Francisco, CA, USA) was used to assist with English‐language prose for clarity and grammar; drafting and debugging statistical R code. All scientific content, data analysis and conclusions were verified by the authors, who take full responsibility for the integrity of the work.

## 3 RESULTS

### 3.1 Specific dynamic action was numerically larger and longer in voluntarily fed fish but sensitive to early disturbance effects

Total SDA energy across the full trial duration (uncorrected for initial post‐entry disturbance) was numerically larger in voluntary‐fed fish (929.5 ± 199.6 mg O_2_ kg^−0^·^79^) than in gavage fish (261.4 ± 52.8 mg O_2_ kg^−0^·^79^; raw *p* = 0.040, Benjamini–Hochberg‐adjusted *p* = 0.140) (Figures 1 and 2; Table 1). Following exclusion of the initial acclimation period, mean SDA decreased in both groups and remained numerically higher in voluntary‐fed fish (647.9 ± 169.7 mg O_2_ kg^−0^·^79^) than in gavage fish (224.4 ± 33.5 mg O_2_ kg^−0^·^79^; Figure 2a), representing an approximately 2.9 fold difference, although this did not reach statistical significance (raw *p* = 0.086, adjusted *p* = 0.151). Within‐group variability was higher in voluntary‐fed fish (CV = 52.4%) than in gavage fish (CV = 29.9%). This variability was largely associated with elevated metabolic rates during the early post‐entry period in non‐anaesthetised (voluntary fed) individuals. Partitioning SDA into digestive and the handling‐associated metabolic cost (early post‐entry elevation) revealed that a substantial proportion of total metabolic elevation in voluntary fish occurred during the initial acclimation phase. The handling‐associated metabolic cost (early post‐entry elevation) was 281.6 ± 38.3 mg O_2_ kg^−0^·^79^ in voluntary fish, compared with 37.0 ± 21.2 mg O_2_ kg^−0^·^79^ in gavage fish (raw *p* = 0.001, adjusted *p* = 0.010; Figure 2b). This indicates that early handling and recovery effects contributed disproportionately to total SDA estimates in voluntary fish.

**FIGURE 1:**
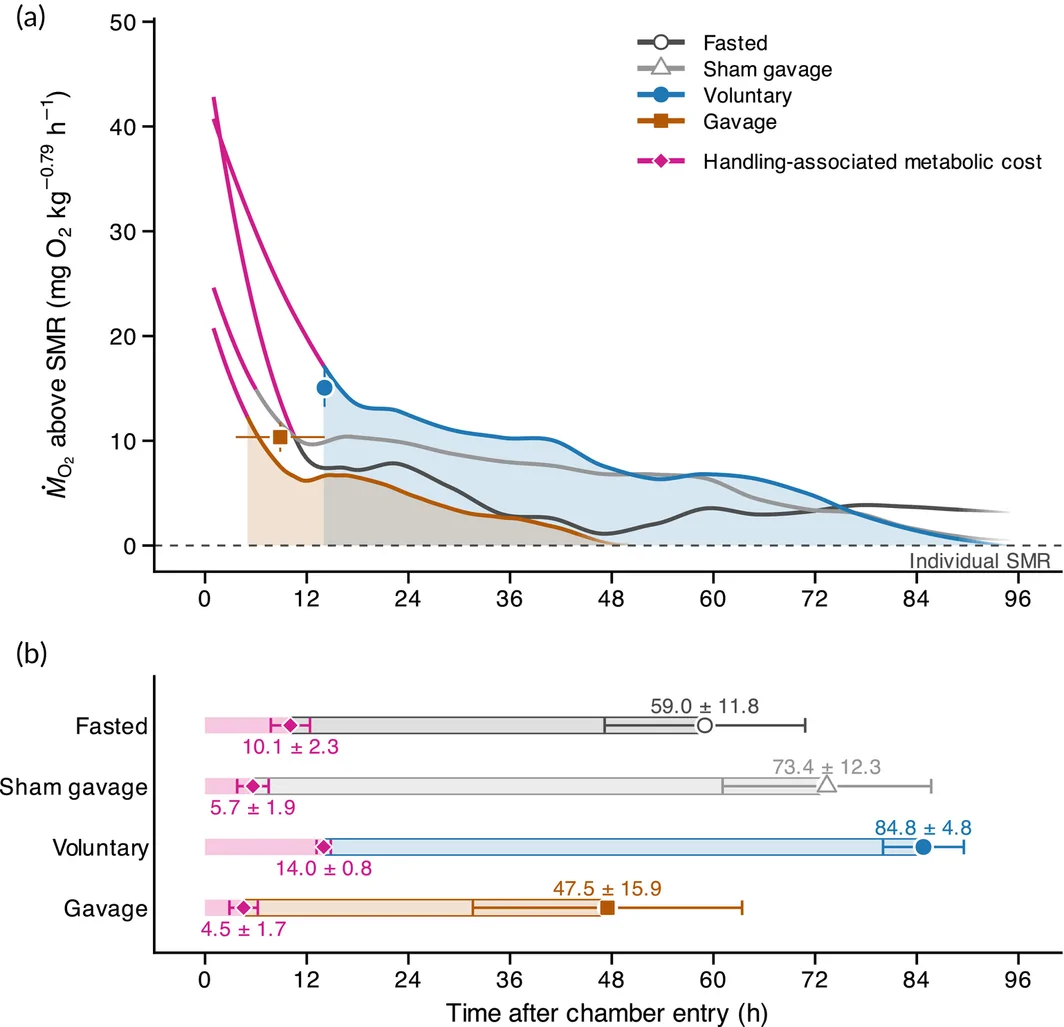
Postprandial oxygen consumption in sand flathead (*Platycephalus bassensis*) following voluntary feeding, intragastric gavage, sham gavage and fasting. (a) Mean ṀO_2_ above each fish's own SMR (dashed line at zero), LOESS‐smoothed for display. Pink sections of each curve denote the handling‐associated period and shaded areas denote SDA. Symbols show peak ṀO_2_ above SMR and time to peak for fed groups (mean ± SEM). (b) Duration of the handling‐associated period (pink) and of SDA, or of the return to SMR in unfed groups (tinted bars). Values are mean ± SEM (h). Time zero denotes entry into the respirometry chamber. *n* = 4 per group.

**FIGURE 2:**
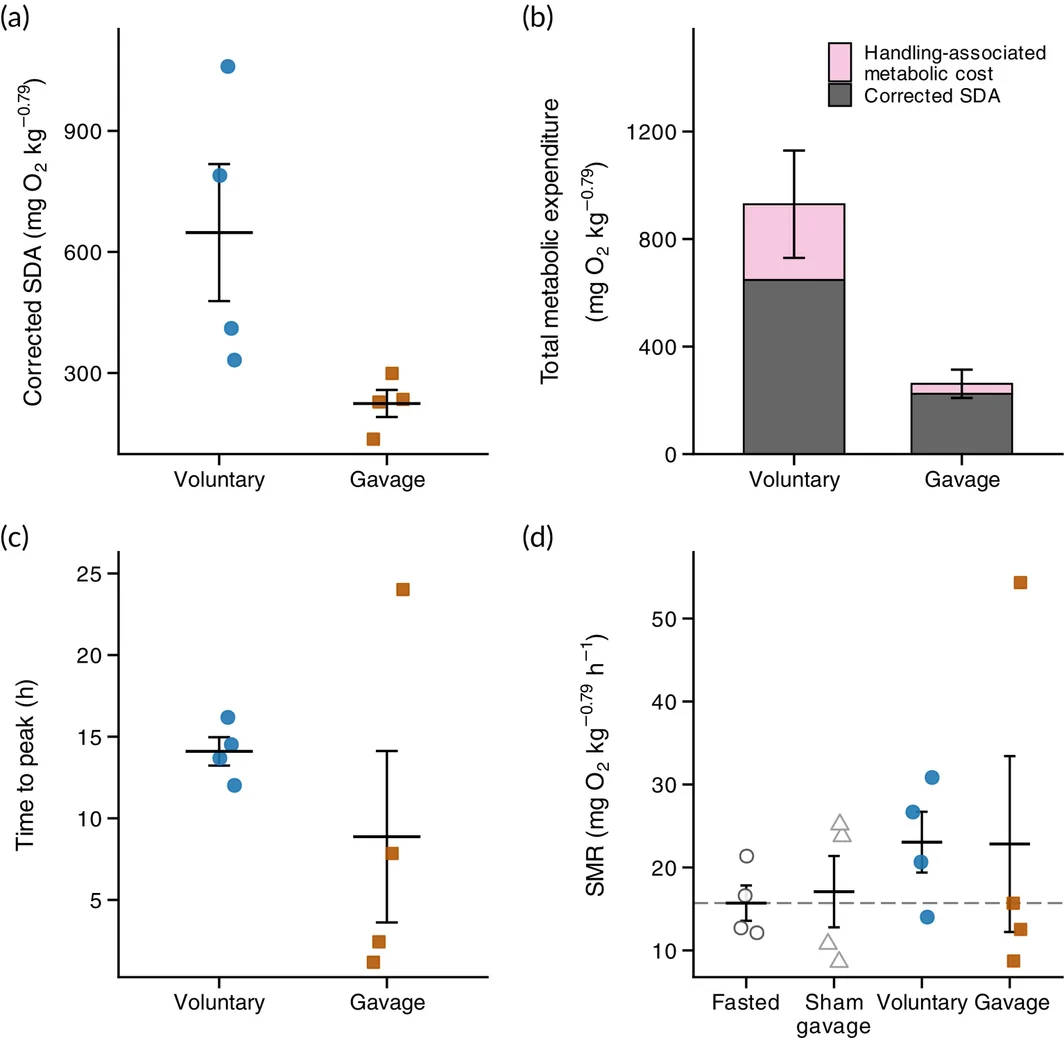
Specific dynamic action (SDA) parameters and standard metabolic rate (SMR) of sand flathead (*Platycephalus bassensis*) following voluntary feeding, intragastric gavage, sham gavage and fasting. (a) Corrected SDA, calculated after exclusion of the initial stabilisation period, for voluntarily fed and gavage‐fed fish. (b) Partitioning of total postprandial metabolic expenditure into corrected SDA (grey) and the handling‐associated metabolic cost (pink), the difference between SDA estimated from the full post‐entry time series and corrected SDA. Bars show group means and error bars show SEM of total expenditure. (c) Time to peak ṀO_2_ for voluntarily fed and gavage‐fed fish. (d) SMR for all four groups, calculated from the final 24 h of each 96 h respirometry trial. The dashed line denotes the mean SMR of the fasted group. In A, C and D, points represent individual fish, horizontal bars show group means and error bars show SEM. *n* = 4 per group.

**TABLE 1:** Standard metabolic rate and specific dynamic action (SDA) parameters in sand flathead (*Platycephalus bassensis*) fed a standardised 1.5% body mass meal either voluntarily or by intragastric gavage.

Parameter	Voluntary (*n* = 4)	Gavage (*n* = 4)	*p*‐value
SMR (mg O_2_ kg^−0^·^79^ h^−1^)	23.05 ± 3.67	22.82 ± 10.60	0.885
SDA (corrected; mg O_2_ kg^−0^·^79^)	647.9 ± 169.7	224.4 ± 33.5	0.151
SDA (uncorrected; mg O_2_ kg^−0^·^79^)	929.5 ± 199.6	261.4 ± 52.8	0.140
Handling‐associated metabolic cost (mg O_2_ kg^−0^·^79^)	281.6 ± 38.3	37.0 ± 21.2	0.010**
SDA Peak (mg O_2_ kg^−0^·^79^ h^−1^)	15.06 ± 1.82	10.35 ± 1.40	0.151
SDA Duration (h)	84.8 ± 4.8	47.5 ± 15.9	0.272
Time to Peak (h)	14.10 ± 0.87	8.87 ± 5.25	0.424

*Note*: Values are mean ± SEM. Uncorrected SDA parameters were calculated from the full time series following chamber entry, corrected parameters exclude the initial stabilisation period, and the handling‐associated metabolic cost is the difference between them. *p*‐values are from pairwise comparisons between feeding methods (Student's or Welch's *t*‐test, or Wilcoxon rank‐sum test, following the assumption checks in Section 2.7), adjusted using the Benjamini–Hochberg false discovery rate procedure. ** indicates *p* < 0.05.

Despite this, peak ṀO_2_ above SMR was numerically higher in voluntary fish (15.06 ± 1.82 mg O_2_ kg^−0^·^79^ h^−1^) than in gavage fish (10.35 ± 1.40 mg O_2_ kg^−0^·^79^ h^−1^), although this difference was not statistically supported (raw *p* = 0.086, adjusted *p* = 0.151; Figure 1a). Similarly, SDA duration was longer in voluntary fish (84.8 ± 4.8 h) than in gavage fish (47.5 ± 15.9 h), but with substantial variation in the gavage group (raw *p* = 0.194, adjusted *p* = 0.272; Figure 1b). TTP ṀO_2_ did not differ between feeding methods (voluntary: 14.10 ± 0.87 h; gavage: 8.87 ± 5.25 h; raw *p* = 0.364, adjusted *p* = 0.424) (Figure 2c).

Terminal SMR, estimated from the final 24 h of each trial, did not differ among groups (all raw *p* ≥ 0.13; all adjusted *p* ≥ 0.80; Figure 2d) The procedural control (sham gavage) did not differ from fasted fish in total metabolic expenditure (*p* = 0.558) or SMR (raw *p* = 0.787, adjusted *p* = 0.885). Post‐mortem observations supported these patterns. Gavage fish showed progressive digestion, with gastric emptying evident in all individuals sampled at ≥12 h. In contrast, fasted and procedural control fish had no detectable gastric contents, confirming complete gastric clearance prior to the trial.

Body mass differed among the four groups (one‐way ANOVA: *F*_3,12_ = 4.46, *p* = 0.025), with voluntarily fed fish heavier than gavage‐fed fish in the Tukey comparison (adjusted *p* = 0.018). Mass‐adjusted sensitivity models retained the direction of the main SDA contrasts: the voluntary‐feeding coefficient remained positive for corrected SDA (*p* = 0.199), uncorrected SDA (*p* = 0.088) and handling‐associated metabolic cost (*p* = 0.005). These small‐sample sensitivity models should be interpreted cautiously. Individual ṀO_2_ traces for each batch are shown in Figure S1.

### 3.2 Postprandial alkaline tide differed in timing and magnitude between voluntary feeding and intragastric gavage

Blood pH and [HCO_3_^−^] between 6 and 24 h post‐feeding were analysed by separate two‐way ANOVAs (Treatment × Time) restricted to the voluntary‐fed and gavage groups, the only treatments with matched time points. For blood pH, the ANOVA revealed a significant Treatment × Time interaction (*F*_2,29_ = 13.79, *p* < 0.001), alongside a main effect of Treatment (*F*_1,29_ = 8.49, *p* = 0.007); the main effect of Time was not significant (*F*_2,29_ = 0.97, *p* = 0.393). In contrast, the ANOVA for [HCO_3_^−^] showed no significant effects (Treatment *F*_1,29_ = 1.39, *p* = 0.247; Time *F*_2,29_ = 0.67, *p* = 0.518; interaction *F*_2,29_ = 1.89, *p* = 0.169). All pairwise comparisons reported below are Tukey HSD post‐hoc contrasts from these models. Voluntary‐fed fish exhibited a clear alkaline tide at 6 h post‐feeding, with blood pH increasing to 7.946 ± 0.034 (*n* = 5) relative to the fasting baseline (7.834 ± 0.036); this corresponded to a large positive effect on both blood pH (Hedges' *g* = +1.66, 95% CI [+0.06, +3.27]) and [HCO_3_^−^] (*g* = +1.71, 95% CI [+0.09, +3.32]) (Figure 3). In contrast, gavage‐fed fish showed no alkaline tide at this time point, with blood pH remaining lower at 7.760 ± 0.012 (*n* = 6). This corresponded to a mean difference of 0.186 pH units between treatments and was highly significant (Tukey HSD: *p* < 0.001; Figure 4a). This divergence in pH was mirrored by differences in bicarbonate dynamics. At 6 h, blood [HCO_3_^−^] was numerically elevated in voluntary‐fed fish (8.97 ± 0.62 mmol L^−1^), whereas gavage‐fed fish showed no comparable increase (7.11 ± 0.83 mmol L^−1^), remaining similar to the procedural baseline (6.00 ± 0.20 mmol L^−1^; Tukey HSD Voluntary vs. Gavage at 6 h: *p* = 0.065) (Figure 4b). In gavage‐fed fish, blood pH at 6 h was also lower than the procedural control (7.803 ± 0.013), suggesting a tendency toward transient acidification (ΔpH = −0.043). Because the procedural control was sampled at only a single time point and could not be incorporated into the two‐way ANOVA, this baseline‐referenced contrast is reported as a descriptive effect size only (Hedges' *g* = −1.33, 95% CI [−2.81, +0.15]; uncorrected Welch/Student's t *p* = 0.052) and was not entered into the primary hypothesis‐testing framework. The consistent direction and large effect size nonetheless suggest a biologically meaningful response that may be underpowered.

**FIGURE 3:**
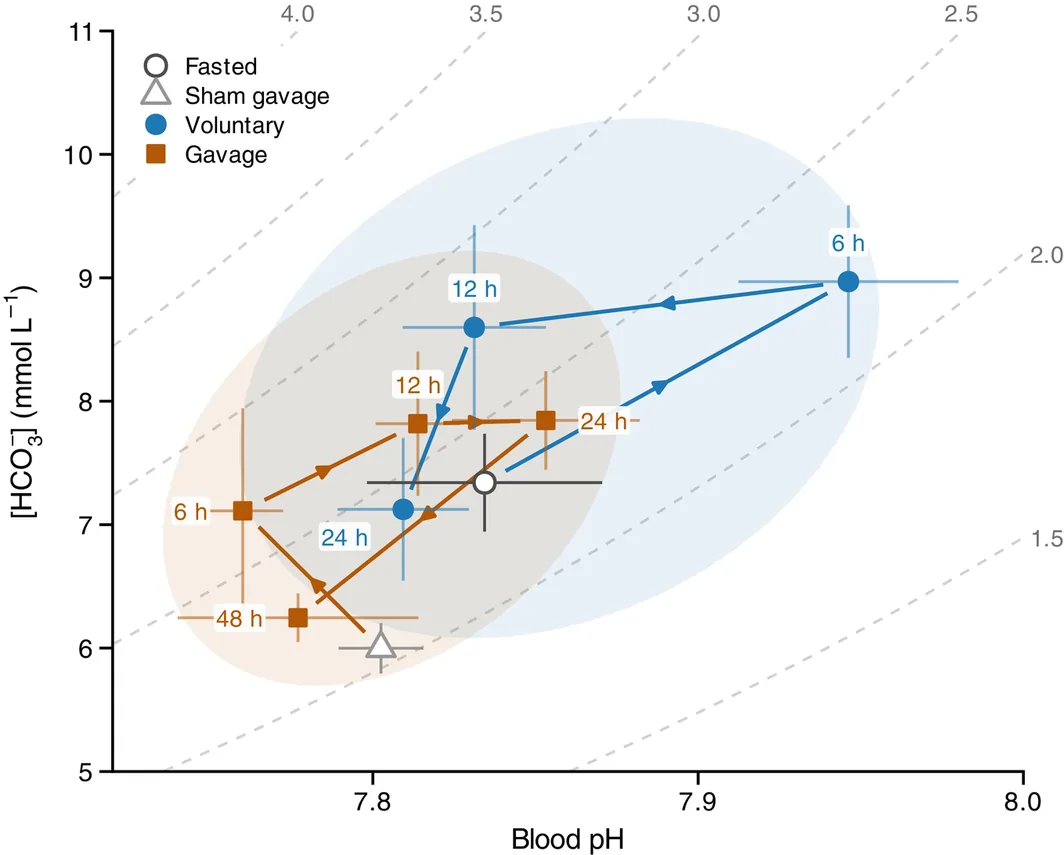
Davenport diagram of whole‐blood pH, bicarbonate concentration ([HCO_3_^−^]) and PCO_2_ in sand flathead (*Platycephalus bassensis*) following voluntary feeding or intragastric gavage *(N = 44; n = 3‐6 per group and timepoint, except gavage at 48 h, n = 2)*. Open symbols show mean ± SEM for the fasted (*n* = 3) and sham gavage (*n* = 4) reference groups. Filled symbols show mean ± SEM for voluntarily fed and gavage‐fed fish at 6, 12, and 24 h post‐feeding, and at 48 h for gavage‐fed fish. Solid lines with arrowheads connect successive time points. Each line starts at its reference group. Shaded ellipses show 50% normal‐theory regions for individual fish in each fed group, pooled across time points. Dashed grey curves are PCO_2_ isopleths from 1.5 to 4.0 mmHg.

**FIGURE 4:**
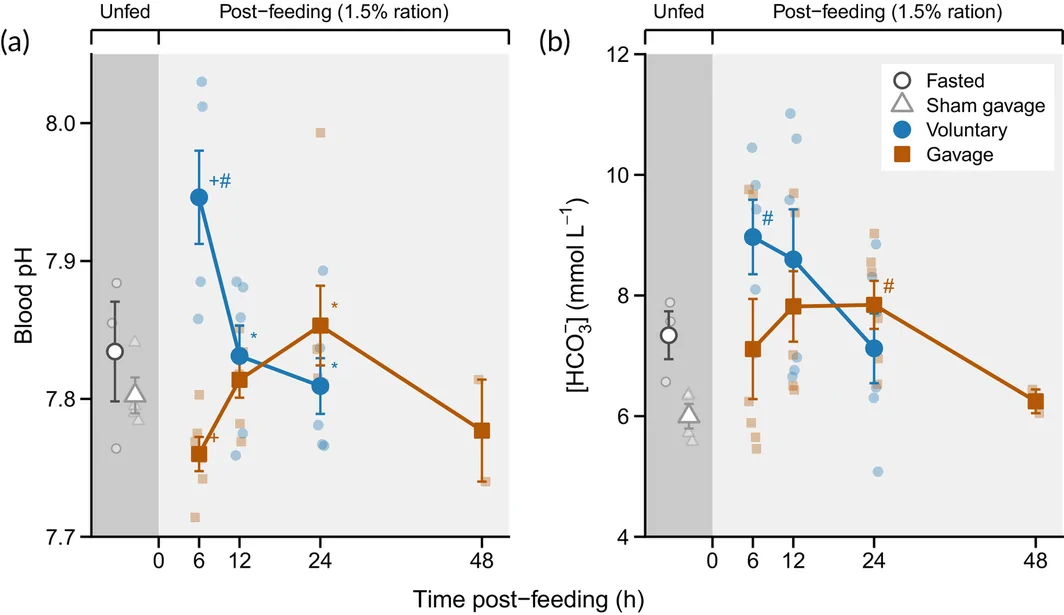
Whole‐blood pH (a) and [HCO_3_^−^] (b) of sand flathead (*Platycephalus bassensis*) following voluntary feeding or intragastric gavage. Faint symbols show individual fish, jittered for clarity and solid symbols show mean ± SEM (*N* = 44; *n* = 3–6 per group and time point, except gavage at 48 h, *n* = 2). Fasted and sham‐gavage fish (sampled 6 h after the procedure with no food) are plotted to the left of time zero as unfed references. *denotes a significant within‐group difference relative to 6 h post‐feeding, and + a significant difference between voluntary and gavage groups at the same time point (Tukey HSD from two‐way ANOVA on voluntary and gavage groups at 6, 12 and 24 h, *p* < 0.05). # denotes a baseline‐referenced effect size of |Hedges' g| ≥ 1 with 95% CI excluding zero, reported descriptively (Methods 2.7; Results 3.2). The gavage 48 h time point was excluded from formal hypothesis tests.

Temperature‐corrected arterial PCO_2_ ranged from 1.91 to 3.75 mmHg across all treatment groups and sampling time points (Figure 5a). Pre‐feeding reference values were 2.34 ± 0.31 mmHg (*n* = 3) in fasted fish and 2.05 ± 0.06 mmHg (*n* = 4) in sham‐gavage fish. Following voluntary feeding, arterial PCO_2_ increased from 2.23 ± 0.23 mmHg (*n* = 5) at 6 h to a peak of 2.76 ± 0.34 mmHg (*n* = 6) at 12 h post‐feeding, before declining to 2.37 ± 0.19 mmHg (*n* = 6) by 24 h. Gavage‐fed fish exhibited elevated PCO_2_ at both 6 h (2.60 ± 0.27 mmHg, *n* = 6) and 12 h (2.60 ± 0.27 mmHg, *n* = 6), with values approaching pre‐feeding levels by 48 h (2.24 ± 0.15 mmHg, *n* = 2). Arterial PCO_2_ was significantly and negatively correlated with blood pH across all groups and time points (Figure 5b; Spearman *ρ* = −0.459, *n* = 44, *p* = 0.002), consistent with the respiratory acid–base response to blood PCO_2_.

**FIGURE 5:**
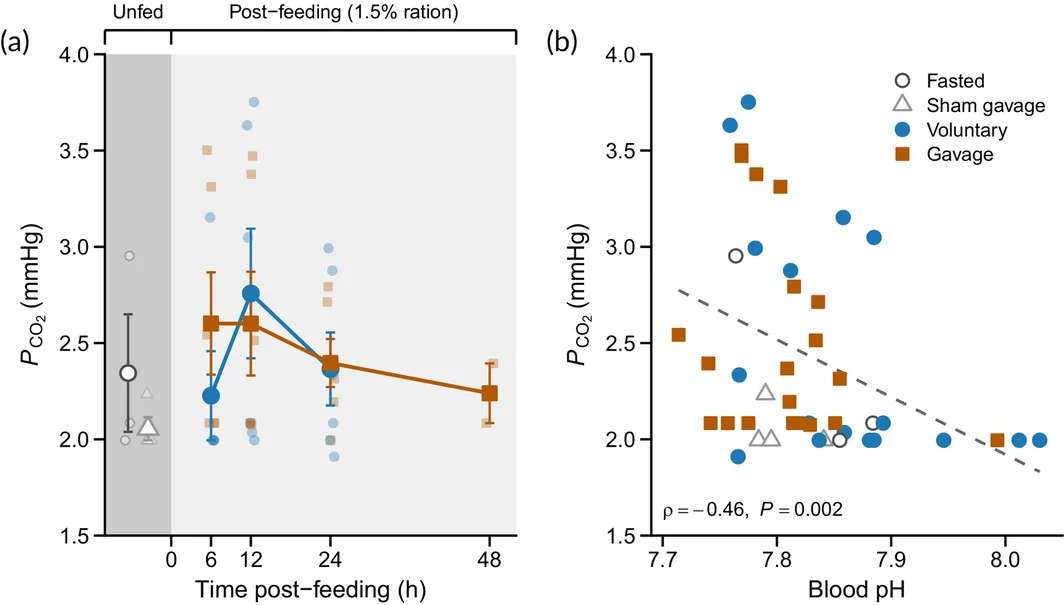
Temperature‐corrected whole‐blood PCO_2_ of sand flathead (*Platycephalus bassensis*) following voluntary feeding, intragastric gavage, sham gavage and fasting. (a) PCO_2_ over time. Faint symbols show individual fish, jittered for clarity and solid symbols show mean ± SEM. Fasted and sham‐gavage fish are plotted to the left of time zero as unfed references. PCO_2_ did not differ between feeding methods or across time (two‐way ANOVA, all *p* > 0.05). (b) Relationship between PCO_2_ and blood pH across all fish (Spearman *ρ* = −0.459, *n* = 44, *p* = 0.002). The dashed line is a least‐squares fit. Values at the analyser's lower detection limit (5.0 mmHg uncorrected, ≈1.91 mmHg after temperature correction) are left‐censored and form the cluster of points at the lower bound.

Following the initial suppression at 6 h, gavage‐fed fish exhibited a delayed and sustained rise in blood [HCO_3_^−^]. At 12 h, [HCO_3_^−^] increased to 7.82 ± 0.58 mmol L^−1^ (baseline‐referenced contrast vs. procedural control: Hedges' *g* = +1.42, 95% CI [−0.08, +2.92]; uncorrected *p* = 0.041, reported descriptively only), and by 24 h the group mean was higher still (7.84 ± 0.40 mmol L^−1^; Hedges' *g* = +2.06, 95% CI [+0.41, +3.71]; uncorrected *p* = 0.008) (Figure 4b). Within the primary two‐way ANOVA, however, the within‐group change from 6 h to 24 h in gavage‐fed fish was not statistically supported for [HCO_3_^−^] (Tukey HSD: *p* = 0.71), and the between‐group contrast with voluntary‐fed fish at 24 h was likewise non‐significant (*p* = 0.44), so this apparent accumulation should be interpreted cautiously and primarily as an effect‐size signal against the depressed procedural baseline. This delayed bicarbonate accumulation was accompanied by a recovery in blood pH within the gavage group from 7.760 ± 0.012 at 6 h to 7.853 ± 0.029 at 24 h (Tukey HSD from the two‐way ANOVA: ΔpH = +0.093, *p* = 0.015), indicating a compensatory acid–base response over time (Figure 4a). In contrast, voluntary‐fed fish showed a transient alkaline tide followed by recovery toward baseline. Blood pH declined from 7.946 ± 0.034 at 6 h to 7.831 ± 0.022 at 12 h and 7.809 ± 0.020 at 24 h (Tukey HSD: 6 h vs. 12 h *p* = 0.004; 6 h vs. 24 h *p* < 0.001; 12 h vs. 24 h *p* = 0.76) (Figure 4a), while [HCO_3_^−^] decreased from 8.97 ± 0.62 mmol L^−1^ at 6 h to 7.12 ± 0.58 mmol L^−1^ at 24 h (Tukey HSD within‐voluntary across time: all *p* ≥ 0.15) (Figure 4b). By 24 h, [HCO_3_^−^] values were similar between voluntary and gavage treatments (Tukey HSD between‐group at 24 h: *p* = 0.44), indicating convergence of acid–base status in the later postprandial period. By 48 h, gavage‐fed fish had returned to values comparable to the fasting baseline (pH: 7.777 ± 0.037; [HCO_3_^−^]: 6.25 ± 0.20 mmol L^−1^; *n* = 2; reported descriptively only and excluded from formal hypothesis tests because of the reduced sample size).

Baseline comparisons indicated that the procedural control provided a reasonable approximation of fasting acid–base status for pH (Welch's *t*‐test *p* = 0.478), but blood [HCO_3_^−^] was numerically lower than in fasted fish (6.00 ± 0.20 vs. 7.34 ± 0.40 mmol L^−1^). Equivalence testing using two one‐sided tests (TOST) did not confirm equivalence within the pre‐specified bounds (±0.05 pH units, max TOST *p* = 0.478; ±1.0 mmol L^−1^ [HCO_3_^−^], max TOST *p* = 0.056), suggesting a small downward shift in baseline [HCO_3_^−^] associated with the intubation procedure. This introduces a conservative bias in comparisons involving gavage‐fed fish, such that the magnitude of bicarbonate accumulation may be underestimated relative to a true fasting baseline.

Blood glucose remained low and stable across all fed groups and time points (range ~ 1.3–1.6 mmol L^−1^), with no difference between voluntary and gavage fish at 6 h (Wilcoxon *p* = 0.391) and no relationship with PCO_2_ (Spearman *ρ* = −0.042, *n* = 36, *p* = 0.806). While glucose does not directly reflect metabolic pathway use, the absence of change suggests that fish were not experiencing a sustained systemic stress response during the measurement period.

### 3.3 Gastrointestinal transit difference

Dissection of fish at 6, 12 and 24 h post‐feeding revealed marked differences in gastrointestinal transit between feeding treatments (Figure 6). At 6 h post‐feeding, voluntarily fed fish retained intact pellets within the stomach with little evidence of onward transit, whereas in gavage‐fed fish the majority of the slurry had already passed through the stomach and entered the intestine. At 12 h, acidified pellet material remained visible in the stomachs of voluntarily fed fish, while the stomachs of gavage‐fed fish were almost empty. At 24 h, acidified digesta was still present in voluntarily fed fish, with the bulk of the material located in the mid‐intestine; in gavage‐fed fish, faecal material had progressed to the distal intestine near the anus.

**FIGURE 6:**
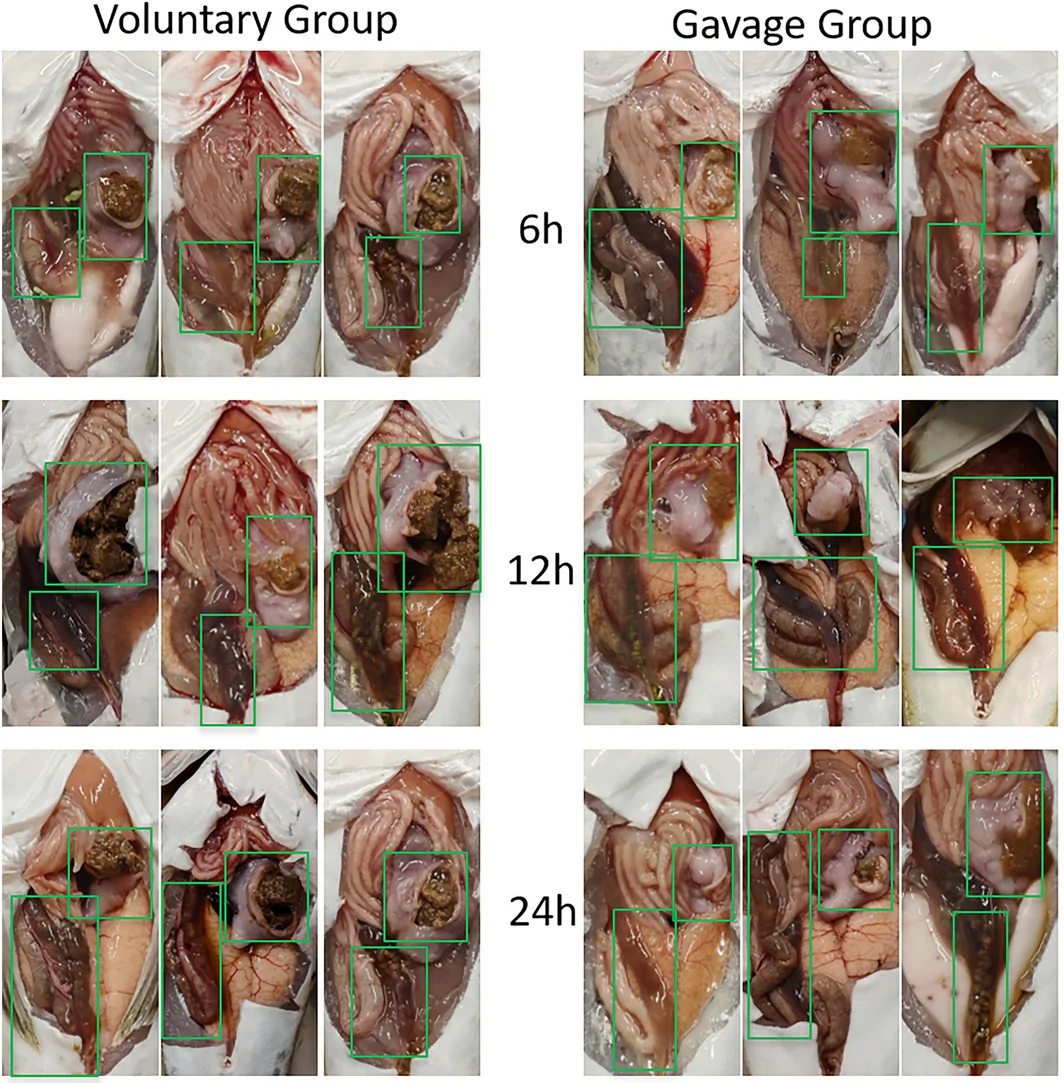
Gastrointestinal transit of intact pellets and homogenised slurry in sand flathead (*Platycephalus bassensis*) at 6, 12 and 24 h post‐feeding. Representative dissection photographs from voluntarily fed fish, which received intact pellets, and gavage‐fed fish, which received an equivalent ration as a homogenised slurry. Three fish are shown per treatment and time point. Green boxes indicate the stomach (upper) and the intestinal region (lower).

## 4 DISCUSSION

This study demonstrates that the method used to deliver a meal alters postprandial responses in *Platycephalus bassensis*. Voluntarily fed fish exhibited a numerically larger and more prolonged SDA response than gavage‐fed fish, although this difference was sensitive to the elevated metabolic rate associated with chamber entry and was no longer statistically supported after correction. Feeding method also altered postprandial acid–base status, with voluntary feeding producing a distinct alkaline tide 6 h after feeding, to our knowledge the first report of this response in *P. bassensis*, whereas gavage suppressed the early alkalosis and was followed by a delayed increase in blood HCO_3_^−^. These patterns suggest that gavage altered the timing and coordination of digestion rather than modifying a single component of the postprandial response.

The physical form of the meal is likely to have contributed substantially to these differences because intact pellets and homogenised slurry differ in particle size, hydration, viscosity and exposed surface area, characteristics that strongly influence gastric retention and evacuation. Gastric emptying in fishes is sensitive to meal consistency and particle structure, with softer or more finely processed meals leaving the stomach more rapidly than intact solids (Bromley, 1994). Minced herring are evacuated faster than whole herring in Atlantic cod (*Gadus morhua* Linnaeus, 1758; dos Santos & Jobling, 1988), and evacuation rate varies with prey structure and with pellet size in other species (Bromley, 1991; Busti et al., 2022). These effects are relevant because gastric digestion not only initiates chemical and mechanical breakdown of the meal but also regulates the progressive delivery of partially processed chyme to the intestine (Bakke et al., 2010). Homogenisation may therefore reduce the period of gastric processing required before evacuation, allowing nutrients to reach the intestine earlier and increasing the temporal overlap between gastric digestion, intestinal processing and absorption. The post‐mortem observations in the present study are consistent with such a shift in digestive timing. At 6 h, most of the gavaged slurry had already passed into the intestine, whereas intact pellets remained within the stomachs of voluntarily fed fish, and by 24 h digesta had progressed further along the intestine following gavage. Although gastric evacuation was not quantified directly, the two treatments clearly differed in the progression of digesta through the tract. This difference provides a plausible explanation for the shorter SDA trajectory observed following gavage, because the longer response in voluntarily fed fish may reflect a more extended period of gastric, intestinal and post‐absorptive activity rather than a fundamentally greater energetic cost of processing and assimilating the meal. Indeed, after the initial chamber‐entry period was excluded, total SDA remained approximately 2.9‐fold greater in voluntarily fed fish, although this difference was not statistically significant, suggesting that the strongest evidence lies in altered temporal dynamics rather than in a definitive difference in total postprandial energy expenditure.

The same alteration in digestive timing provides a useful framework for interpreting the acid–base responses. Voluntarily fed *P. bassensis* showed a clear increase in blood pH 6 h after feeding, from 7.834 to 7.946, corresponding to a 22.8% reduction in circulating H^+^ concentration and a response consistent with the postprandial alkaline tide generated during gastric acid secretion. We base this interpretation on the directly measured pH signal, because the precision of the i‐STAT analyser is reduced at the low PCO_2_ values characteristic of water‐breathing fishes and this uncertainty propagates through the Henderson–Hasselbalch calculation into estimated HCO_3_^−^ (Harter et al., 2014; see Section 2.6). Calculated HCO_3_^−^ accordingly differed less consistently between treatments than pH, although the direction of the two responses was concordant throughout. Among fishes, the alkaline tide generally develops within the first several hours after feeding and is commonly accompanied by an increase in circulating HCO_3_^−^ (Wood, 2019), placing the timing and magnitude observed here within the range reported for other gastric species. In contrast, gavage‐fed fish showed no detectable alkaline tide at 6 h and instead exhibited an elevation in blood HCO_3_^−^ at 24 h. This later response indicates that the systemic acid–base disturbance was delayed rather than entirely absent and suggests that gavage altered the temporal relationship among the processes generating and compensating for the alkaline load.

Such an effect could arise if the more rapid passage of slurry from the stomach increased the overlap between gastric acid secretion, intestinal neutralisation and subsequent acid–base compensation. The alkaline tide measured in blood represents the net outcome of several simultaneously operating processes rather than a direct measure of gastric H^+^ secretion alone, and changes in their relative timing can therefore alter the magnitude of the systemic signal (Bucking & Wood, 2008; Wood, 2019; Wood et al., 2005). Earlier delivery of fluid digesta to the intestine could, for example, bring intestinal bicarbonate secretion and other compensatory processes into closer temporal alignment with gastric secretion, thereby attenuating the early rise in systemic pH even if substantial acid secretion were occurring. The persistence of elevated HCO_3_^−^ at 24 h, despite post‐mortem evidence that much of the meal had already left the stomach, is also consistent with a lag between generation of the postprandial alkaline load and its subsequent clearance. Recovery from acid–base disturbances in teleosts depends on branchial ion‐transport pathways involving H^+^‐ATPases, Na^+^/H^+^ exchangers and Cl^−^/HCO_3_^−^ exchange (Claiborne et al., 2002; Evans et al., 2005), and adjustment of these systems occurs over hours rather than minutes (Perry & Gilmour, 2006). Comparable timescales have been reported in other fishes, with branchial base excretion peaking 12–24 h after feeding in dogfish (Wood et al., 2005) and complete resolution of the alkaline tide requiring 24–72 h in rainbow trout depending on feeding method (Bucking & Wood, 2008; Cooper & Wilson, 2008). The return of blood pH and HCO_3_^−^ toward fasting values by 48 h in *P. bassensis* is therefore consistent with the prolonged recovery expected following a postprandial acid–base disturbance.

The suppression of the early alkaline tide following gavage contrasts with the response reported by Cooper and Wilson (2008) in rainbow trout (*Oncorhynchus mykiss* Walbaum, 1792), where force‐fed fish developed a larger and more prolonged alkaline tide than voluntarily fed individuals receiving the same ration. Cooper and Wilson proposed that bypassing the cephalic phase of digestion removed anticipatory compensatory responses that would normally buffer the systemic effects of gastric acid secretion, such that involuntary delivery of a 1% ration produced an acid–base disturbance comparable to voluntary consumption of a substantially larger meal. Sensory cues associated with food detection, capture and swallowing do initiate neural and endocrine responses before nutrients reach the gut (Fänge & Grove, 1979; Power & Schulkin, 2008; Rønnestad et al., 2017), and gavage bypasses much of this anticipatory sequence. The opposing direction of the response observed here, however, indicates that loss of cephalic stimulation alone cannot explain the effects of feeding method across studies, and instead emphasises that gavage is not a single physiological manipulation with effects that remain constant across experimental contexts. For instance, Cooper and Wilson delivered that ration to unanaesthetised fish through an indwelling oesophageal catheter, whereas the present study delivered a larger slurry meal directly into the stomach by syringe under benzocaine anaesthesia. The studies therefore differed simultaneously in species, ration size, route of delivery, anaesthetic exposure and aspects of acid–base measurement, each of which could influence the magnitude or timing of the postprandial response. Species‐specific differences in gastric evacuation, acid secretion or branchial compensation may also contribute. The acid–base consequences of force feeding are thus context dependent, emerging from the interaction between species biology and the broader experimental procedure.

Oral gavage is typically performed under sedation, and in Australia it is treated as a veterinary intervention requiring appropriate training (Collymore et al., 2013). Indwelling catheters avoid anaesthesia but introduce additional surgical manipulation (Cooper & Wilson, 2008). Our objective was therefore to compare voluntary feeding with gavage as it is operationally applied, rather than to isolate mechanical deposition of food from every associated procedural effect. Benzocaine can produce a prolonged cortisol response lasting several hours (Zahl et al., 2012), and deep anaesthesia has altered oxygen consumption for at least 4 h after apparent recovery in goldfish (*Carassius auratus* Linnaeus, 1758; Herrera‐Castillo et al., 2024). These effects could contribute to differences in early postprandial metabolism; however, a simple respiratory explanation for the suppressed alkaline tide appears unlikely because blood sampling was conducted under comparable anaesthetic conditions with continuous gill irrigation to minimise artefactual CO_2_ accumulation (Davison et al., 2023), and PCO_2_ did not differ significantly between feeding treatments. Anaesthesia may instead have influenced gastrointestinal function directly, as anaesthetic agents can modify gut motility in other vertebrates (Qualls‐Creekmore et al., 2010), while general anaesthesia has been shown to suppress gastrin‐stimulated gastric acid secretion in mammals (Norlén et al., 2000). Comparable effects remain poorly characterised in fishes, but a transient alteration in gastric secretion or motility following benzocaine exposure could have further modified the timing of digestion initiated by delivery of the homogenised meal, thereby contributing to the weaker early alkaline tide and its delayed appearance. Because these effects cannot be separated experimentally from meal form, route of delivery and the absence of normal feeding behaviour, the observed response is best interpreted as the integrated physiological consequence of the gavage procedure rather than evidence for any single causal mechanism.

These findings highlight both the experimental value and the physiological limitations of gavage as a method for studying digestion in fishes. Gavage provides precise control over ration size and feeding time and, in the present study, was associated with lower within‐treatment variability in estimates of SDA, which may be advantageous in experiments where voluntary intake is difficult to standardise. Such control does not make gavage physiologically equivalent to voluntary feeding, because the procedure simultaneously alters meal structure, gastrointestinal transit, anticipatory feeding responses and anaesthetic exposure. These differences provide important context when interpreting SDA magnitude and duration, selecting postprandial sampling intervals, or comparing acid–base responses among studies using different feeding protocols. Separating these components will require standardised anaesthetic and transfer conditions across feeding treatments, quantitative measurement of gastric evacuation for intact and homogenised meals, and targeted assessment of anaesthetic effects on gastric secretion, gut motility and postprandial metabolism.

## 5 CONCLUSIONS

The method used to deliver a meal altered postprandial physiology in *P. bassensis*. Voluntary feeding elicited a distinct alkaline tide 6 h after feeding, the first documentation of this response in the species, whereas gavage suppressed the early alkalosis and produced a delayed rise in blood HCO_3_^−^. Voluntarily fed fish also showed a larger and longer SDA response, although this difference was not statistically supported after correction for chamber‐entry disturbance. The alkaline tide has now been documented across elasmobranchs (Wood et al., 2005, 2007), salmonids (Bucking & Wood, 2008; Cooper & Wilson, 2008; Goodrich, Berry, et al., 2022), silurids (Li et al., 2010), latids (Goodrich, Wilson, et al., 2022) and a platycephalid, further supporting its broad occurrence among gastric fishes. The parallel changes in SDA and acid–base status suggest that gavage altered the timing and coordination of digestion through the combined effects of faster transit of a homogenised meal, bypassed feeding‐related signalling and anaesthetic exposure. Gavage remains valuable for controlling ration size and delivery, but it should not be treated as a physiologically neutral substitute for voluntary feeding, and meal form, feeding method and associated procedural effects warrant explicit consideration when comparing postprandial responses across studies.

## AUTHOR CONTRIBUTIONS

*Conceptualisation*: Harriet R. Goodrich, Barrett W. Wolfe; *Methodology*: Junlin Chen, Harriet R. Goodrich, Barrett W. Wolfe; *Investigation*: Junlin Chen; *Formal analysis*: Junlin Chen; *Writing—original draft*: Junlin Chen; *Writing—review & editing*: Junlin Chen, Harriet R. Goodrich, Barrett W. Wolfe; *Supervision*: Harriet R. Goodrich, Barrett W. Wolfe.

## FUNDING INFORMATION

This work was supported by the Institute for Marine and Antarctic Studies.

## CONFLICT OF INTEREST STATEMENT

The authors declare no conflicts of interest.

## Supporting information


**Figure S1.** Time series of oxygen consumption rate (MO_2_) in sand flathead (*P. bassensis*) following feeding. Individual MO_2_ time courses across batches (Batch 1–5), with each line representing one individual fish; colours denote treatment group (fasted, dark grey; sham gavage, light grey; voluntary, blue; gavage, brown).
**Table S1.** Summary of studies measuring postprandial fish physiology by taxon and meal delivery (voluntary feeding or intragastric gavage). Counts in each cell are numbers of studies using each method for each species, followed by superscript reference numbers corresponding to reference numbers in Table S2.
**Table S2.** Literature survey of primary research on fish postprandial physiology (1972–2025), classified by method of meal delivery. Studies were identified through Google Scholar and reference‐list searches and were included if they experimentally fed fish, measured a postprandial metabolic, acid–base or digestive variable, and explicitly stated the feeding method. For each study, the table reports the species or taxonomic group, the method of meal delivery (voluntary feeding or intragastric gavage), the year of publication and the full reference. See Materials and Methods for full inclusion and exclusion criteria.

## Data Availability

All data and details of resources can be found within the article and its supporting information.

## References

[jfb70678-bib-0001] Anderson, C. , & Campbell, G. (1988). Immunohistochemical study of 5‐HT‐containing neurons in the teleost intestine: Relationship to the presence of enterochromaffin cells. Cell and Tissue Research, 254(3), 553–559. 10.1007/BF00226505 3233650

[jfb70678-bib-0002] Ashwood, E. R. , Kost, G. , & Kenny, M. (1983). Temperature correction of blood‐gas and pH measurements. Clinical Chemistry, 29(11), 1877–1885. 6354511

[jfb70678-bib-0003] Bakke, A. M. , Glover, C. , & Krogdahl, Å. (2010). Feeding, digestion and absorption of nutrients. In Fish physiology (Vol. 30, pp. 57–110). Academic Press.

[jfb70678-bib-0004] Barnes, L. M. , Leclerc, M. , Gray, C. A. , & Williamson, J. E. (2011). Dietary niche differentiation of five sympatric species of Platycephalidae. Environmental Biology of Fishes, 90(4), 429–441. 10.1007/s10641-010-9752-4

[jfb70678-bib-0005] Beamish, F. W. H. (1974). Apparent specific dynamic action of largemouth bass, *Micropterus salmoides* . Journal of the Fisheries Research Board of Canada, 31, 1763–1769. 10.1139/f74-224

[jfb70678-bib-0006] Belal, I. E. H. (2005). A review of some fish nutrition methodologies. Bioresource Technology, 96(4), 395–402. 10.1016/j.biortech.2003.11.030 15491819

[jfb70678-bib-0007] Boutilier, R. G. , Heming, T. A. , & Iwama, G. K. (1984). Physicochemical parameters for use in fish respiratory physiology. In Fish physiology (Vol. 10, pp. 403–430). Academic Press.

[jfb70678-bib-0008] Bromley, P. J. (1991). Gastric evacuation in cod (Gadus morhua L.) (Vol. 193, pp. 93–98). ICES Marine Science Symposium.

[jfb70678-bib-0009] Bromley, P. J. (1994). The role of gastric evacuation experiments in quantifying the feeding rates of predatory fish. Reviews in Fish Biology and Fisheries, 4, 36–66. 10.1007/BF00043260

[jfb70678-bib-0010] Bucking, C. , & Wood, C. M. (2008). The alkaline tide and ammonia excretion after voluntary feeding in freshwater rainbow trout. Journal of Experimental Biology, 211(15), 2533–2541. 10.1242/jeb.015610 18626089

[jfb70678-bib-0011] Busti, S. , Bonaldo, A. , Diana, A. , Perfetti, S. , Viroli, C. , Fontanillas, R. , Eriksen, T. B. , Gatta, P. P. , & Parma, L. (2022). The incidence of different pellet sw on growth, gut evacuation, feed digestibility and feed waste in gilthead sea bream (*Sparus aurata*). Aquaculture, 555, 738204.

[jfb70678-bib-0012] Chabot, D. , Steffensen, J. F. , & Farrell, A. P. (2016). The determination of standard metabolic rate in fishes. Journal of Fish Biology, 88(1), 81–121. 10.1111/jfb.12845 26768973

[jfb70678-bib-0013] Claiborne, J. B. , Edwards, S. L. , & Morrison‐Shetlar, A. I. (2002). Acid‐base regulation in fishes: Cellular and molecular mechanisms. Journal of Experimental Zoology, 293(3), 302–319. 10.1002/jez.10125 12115903

[jfb70678-bib-0014] Clarke, A. , & Johnston, N. M. (1999). Scaling of metabolic rate with body mass and temperature in teleost fish. Journal of Animal Ecology, 68(5), 893–905. 10.1046/j.1365-2656.1999.00337.x

[jfb70678-bib-0015] Collymore, C. , Rasmussen, S. , & Tolwani, R. J. (2013). Gavaging adult zebrafish. Journal of Visualized Experiments, 78, 50691. 10.3791/50691 PMC3855001 23962977

[jfb70678-bib-0016] Cooper, C. A. , & Wilson, R. W. (2008). Post‐prandial alkaline tide in freshwater rainbow trout: Effects of meal anticipation on recovery from acid‐base and ion regulatory disturbances. Journal of Experimental Biology, 211(15), 2542–2550. 10.1242/jeb.015586 18626090

[jfb70678-bib-0017] Damsgaard, C. , Gam, L. T. H. , Tuong, D. D. , Thinh, P. V. , Huong Thanh, D. T. , Wang, T. , & Bayley, M. (2015). High capacity for extracellular acid–base regulation in the air‐breathing fish *Pangasianodon hypophthalmus* . Journal of Experimental Biology, 218(9), 1290–1294. 10.1242/jeb.117671 25792754

[jfb70678-bib-0018] Davison, W. G. , Cooper, C. A. , Sloman, K. A. , & Wilson, R. W. (2023). A method for measuring meaningful physiological variables in fish blood without surgical cannulation. Scientific Reports, 13, 899. 10.1038/s41598-023-28061-w PMC9845352 36650204

[jfb70678-bib-0019] Dockray, G. J. , Varro, A. , Dimaline, R. , & Wang, T. (2001). The gastrins: Their production and biological activities. Annual Review of Physiology, 63, 119–139. 10.1146/annurev.physiol.63.1.119 11181951

[jfb70678-bib-0020] dos Santos, J. , & Jobling, M. (1988). Gastric emptying in cod, *Gadus morhua* L.: Effects of food particle size and dietary energy content. Journal of Fish Biology, 33(4), 511–516.

[jfb70678-bib-0021] Evans, D. H. , Piermarini, P. M. , & Choe, K. P. (2005). The multifunctional fish gill: Dominant site of gas exchange, osmoregulation, acid‐base regulation, and excretion of nitrogenous waste. Physiological Reviews, 85(1), 97–177. 10.1152/physrev.00050.2003 15618479

[jfb70678-bib-0022] Fänge, R. , & Grove, D. (1979). Digestion. In W. S. Hoar , D. J. Randall , & J. R. Brett (Eds.), Fish physiology (pp. 162–260). Academic Press.

[jfb70678-bib-0023] Gam, L. T. H. , Jensen, F. B. , Damsgaard, C. , Huong, D. T. T. , Phuong, N. T. , & Bayley, M. (2017). Extreme nitrite tolerance in the clown knifefish *Chitala ornata* is linked to up‐regulation of methaemoglobin reductase activity. Aquatic Toxicology, 187, 9–17. 10.1016/j.aquatox.2017.03.013 28351760

[jfb70678-bib-0024] Garber, K. J. (1983). Effect of fish size, meal size and dietary moisture on gastric evacuation of pelleted diets by yellow perch, *Perca flavescens* . Aquaculture, 34, 41–49. 10.1016/0044-8486(83)90290-9

[jfb70678-bib-0025] Goodrich, H. R. , Berry, A. A. , Montgomery, D. W. , Davison, W. G. , & Wilson, R. W. (2022). Fish feeds supplemented with calcium‐based buffering minerals decrease stomach acidity, increase the blood alkaline tide and cost more to digest. Scientific Reports, 12(1), 18468. 10.1038/s41598-022-22496-3 PMC9630376 36323724

[jfb70678-bib-0026] Goodrich, H. R. , Rossiter‐Hill, F. , Audzijonyte, A. , Wolfe, B. W. , Breslin, R. , & Tracey, S. R. (2026). Phenotypic divergence of sand flathead (*Platycephalus bassensis*) between heavily and lightly fished regions in Tasmania, Australia. Conservation Physiology, 14(1), coag001. 10.1093/conphys/coag001 PMC12885885 41676003

[jfb70678-bib-0027] Goodrich, H. R. , Wilson, R. W. , Smullen, R. , Barnes, A. C. , & Franklin, C. E. (2022). Acidified fish feeds reduce the energetic and physiological costs of digestion in juvenile barramundi (*Lates calcarifer*). Aquaculture, 546, 737400. 10.1016/j.aquaculture.2021.737400

[jfb70678-bib-0028] Goodrich, H. R. , Wood, C. M. , Wilson, R. W. , Clark, T. D. , Last, K. B. , & Wang, T. (2024). Specific dynamic action: The energy cost of digestion or growth? Journal of Experimental Biology, 227(7), jeb246722. 10.1242/jeb.246722 38533751

[jfb70678-bib-0029] Grove, D. J. , & Campbell, G. (1979). Effects of extrinsic nerve stimulation on the stomach of the flathead, *Platycephalus bassensis* Cuvier and Valenciennes. Comparative Biochemistry and Physiology Part C: Comparative Pharmacology, 63(2), 373–380. 10.1016/0306-4492(79)90089-3 40753

[jfb70678-bib-0030] Grove, D. J. , Loizides, L. G. , & Nott, J. (1978). Satiation amount, frequency of feeding and gastric emptying rate in *Salmo gairdneri* . Journal of Fish Biology, 12(5), 507–516. 10.1111/j.1095-8649.1978.tb04195.x

[jfb70678-bib-0031] Harter, T. S. , Shartau, R. B. , Brauner, C. J. , & Farrell, A. P. (2014). Validation of the i‐STAT system for the analysis of blood parameters in fish. Conservation Physiology, 2, cou037. 10.1093/conphys/cou037 PMC4732487 27293658

[jfb70678-bib-0032] Herrera‐Castillo, L. , Vallejo‐Palma, G. , Saiz, N. , Sánchez‐Jiménez, A. , Isorna, E. , Ruiz‐Jarabo, I. , & de Pedro, N. (2024). Metabolic rate of goldfish (*Carassius auratus*) in the face of common aquaculture challenges. Biology, 13(10), 804. 10.3390/biology13100804 PMC11504095 39452113

[jfb70678-bib-0033] Iwama, G. K. , McGeer, J. C. , & Pawluk, M. P. (1989). The effects of five fish anaesthetics on acid–base balance, hematocrit, blood gases, cortisol, and adrenaline in rainbow trout. Canadian Journal of Zoology, 67(8), 2065–2073. 10.1139/z89-294

[jfb70678-bib-0034] Jobling, M. (1981). The influences of feeding on the metabolic rate of fishes: A short review. Journal of Fish Biology, 18(4), 385–400. 10.1111/j.1095-8649.1981.tb03780.x

[jfb70678-bib-0035] Jobling, M. (1987). Influences of food particle size and dietary energy content on patterns of gastric evacuation in fish: Test of a physiological model of gastric emptying. Journal of Fish Biology, 30, 299–314. 10.1111/j.1095-8649.1987.tb05754.x

[jfb70678-bib-0036] Jobling, M. (1994). Fish bioenergetics. Chapman & Hall.

[jfb70678-bib-0037] Jobling, M. , & Davies, P. S. (1980). Effects of feeding on metabolic rate, and the specific dynamic action in plaice, *Pleuronectes platessa* L. Journal of Fish Biology, 16, 629–638. 10.1111/j.1095-8649.1980.tb03742.x

[jfb70678-bib-0038] Jutfelt, F. , Norin, T. , Åsheim, E. R. , Rowsey, L. E. , Andreassen, A. H. , Morgan, R. , Clark, T. D. , & Speers‐Roesch, B. (2021). 'Aerobic scope protection' reduces ectotherm growth under warming. Functional Ecology, 35(7), 1397–1407. 10.1111/1365-2435.13811

[jfb70678-bib-0039] Killen, S. S. , Atkinson, D. , & Glazier, D. S. (2010). The intraspecific scaling of metabolic rate with body mass in fishes depends on lifestyle and temperature. Ecology Letters, 13(2), 184–193. 10.1111/j.1461-0248.2009.01415.x 20059525

[jfb70678-bib-0040] Killen, S. S. , Christensen, E. A. F. , Cortese, D. , Závorka, L. , Norin, T. , Cotgrove, L. , Crespel, A. , Munson, A. , Nati, J. J. H. , Papatheodoulou, M. , & McKenzie, D. J. (2021). Guidelines for reporting methods to estimate metabolic rates by aquatic intermittent‐flow respirometry. Journal of Experimental Biology, 224(18), jeb242522. 10.1242/jeb.242522 PMC8467026 34520540

[jfb70678-bib-0041] Li, K.‐G. , Cao, Z.‐D. , Peng, J.‐L. , & Fu, S.‐J. (2010). The metabolic responses and acid‐base status after feeding, exhaustive exercise, and both feeding and exhaustive exercise in Chinese catfish (*Silurus asotus* Linnaeus). Journal of Comparative Physiology B, 180(5), 661–671. 10.1007/s00360-010-0443-4 20127095

[jfb70678-bib-0042] Mandelman, J. W. , & Skomal, G. B. (2009). Differential sensitivity to capture stress assessed by blood acid‐base status in five carcharhinid sharks. Journal of Comparative Physiology B, 179(3), 267–277. 10.1007/s00360-008-0306-4 18846381

[jfb70678-bib-0043] McCue, M. D. (2006). Specific dynamic action: A century of investigation. Comparative Biochemistry and Physiology Part A: Molecular & Integrative Physiology, 144(4), 381–394. 10.1016/j.cbpa.2006.03.011 16716621

[jfb70678-bib-0044] Moffatt, K. , Rossi, M. , Park, E. , Svendsen, J. C. , & Wilson, J. M. (2022). Inhibition of gastric acid secretion with omeprazole affects fish specific dynamic action and growth rate: Implications for the development of phenotypic stomach loss. Frontiers in Physiology, 13, 966447. 10.3389/fphys.2022.966447 PMC9552000 36237533

[jfb70678-bib-0045] Niv, Y. , & Fraser, G. M. (2002). The alkaline tide phenomenon. Journal of Clinical Gastroenterology, 35(1), 5–8. 10.1097/00004836-200207000-00003 12080218

[jfb70678-bib-0046] Norin, T. , & Gamperl, A. K. (2018). Metabolic scaling of individuals vs. populations: Evidence for variation in scaling exponents among different intraspecific groups. Functional Ecology, 32(4), 1084–1094.

[jfb70678-bib-0047] Norlén, P. , Kitano, M. , Lindström, E. , & Håkanson, R. (2000). Anaesthetic agents inhibit gastrin‐stimulated but not basal histamine release from rat stomach ECL cells. British Journal of Pharmacology, 130(4), 725–730. 10.1038/sj.bjp.0703347 PMC1572120 10864877

[jfb70678-bib-0048] Nunn, J. F. , Bergman, N. A. , Bunatyan, A. , & Coleman, A. J. (1965). Temperature coefficients for PCO_2_ and PO_2_ of blood in vitro. Journal of Applied Physiology, 20(1), 23–26. 10.1152/jappl.1965.20.1.23 14257555

[jfb70678-bib-0049] Parks, S. K. , Tresguerres, M. , & Goss, G. G. (2007). Interactions between Na^+^ channels and Na^+^‐HCO_3_ ^−^ cotransporters in the freshwater fish gill MR cell: A model for transepithelial Na^+^ uptake. American Journal of Physiology‐Cell Physiology, 292(2), C935–C944. 10.1152/ajpcell.00604.2005 17005600

[jfb70678-bib-0050] Perry, S. F. , & Gilmour, K. M. (2006). Acid‐base balance and CO_2_ excretion in fish: Unanswered questions and emerging models. Respiratory Physiology & Neurobiology, 154(1–2), 199–215. 10.1016/j.resp.2006.04.010 16777496

[jfb70678-bib-0051] Power, M. L. , & Schulkin, J. (2008). Anticipatory physiological regulation in feeding biology: Cephalic phase responses. Appetite, 50(2–3), 194–206. 10.1016/j.appet.2007.10.006 PMC2297467 18045735

[jfb70678-bib-0052] Qualls‐Creekmore, E. , Tong, M. , & Holmes, G. (2010). Gastric emptying of enterally administered liquid meal in conscious rats and during sustained anaesthesia. Neurogastroenterology and Motility, 22(2), 181–186. 10.1111/j.1365-2982.2009.01393.x PMC2806511 19735361

[jfb70678-bib-0067] R Core Team . (2025). R: A Language and Environment for Statistical Computing. R Foundation for Statistical Computing. https://www.R‐project.org/

[jfb70678-bib-0053] Rønnestad, I. , Gomes, A. , Murashita, K. , Angotzi, R. , Jönsson, E. , & Volkoff, H. (2017). Appetite‐controlling endocrine systems in teleosts. Frontiers in Endocrinology, 8, 73. 10.3389/fendo.2017.00073 PMC5394176 28458653

[jfb70678-bib-0054] Secor, S. M. (2003). Gastric function and its contribution to the postprandial metabolic response of the Burmese python *Python molurus* . Journal of Experimental Biology, 206(10), 1621–1630. 10.1242/jeb.00300 12682094

[jfb70678-bib-0055] Secor, S. M. (2009). Specific dynamic action: A review of the postprandial metabolic response. Journal of Comparative Physiology B, 179(1), 1–56. 10.1007/s00360-008-0301-x 18597096

[jfb70678-bib-0056] Svendsen, M. B. S. , Bushnell, P. G. , & Steffensen, J. F. (2016). Design and setup of intermittent‐flow respirometry system for aquatic organisms. Journal of Fish Biology, 88(1), 26–50. 10.1111/jfb.12797 26603018

[jfb70678-bib-0057] Svendsen, M. B. S. , Bushnell, P. G. , & Steffensen, J. F. (2019). AquaResp 3 . (Version V3.0). Zenodo 10.5281/zenodo.2584015

[jfb70678-bib-0058] Tandler, A. , & Beamish, F. W. H. (1980). Specific dynamic action and diet in largemouth bass, *Micropterus salmoides* (Lacépède). Journal of Nutrition, 110(4), 750–764. 10.1093/jn/110.4.750 7365544

[jfb70678-bib-0059] Taylor, J. R. , & Grosell, M. (2009). The intestinal response to feeding in seawater gulf toadfish, *Opsanus beta*, includes elevated base secretion and increased epithelial oxygen consumption. Journal of Experimental Biology, 212(23), 3873–3881. 10.1242/jeb.034579 19915130

[jfb70678-bib-0060] Tresguerres, M. , Parks, S. K. , Wood, C. M. , & Goss, G. G. (2007). V‐H^+^‐ATPase translocation during blood alkalosis in dogfish gills: Interaction with carbonic anhydrase and involvement in the postfeeding alkaline tide. American Journal of Physiology. Regulatory, Integrative and Comparative Physiology, 292(5), R2012–R2019. 10.1152/ajpregu.00814.2006 17204588

[jfb70678-bib-0061] Wolfe, B. W. (2026). Flatheadresp: Analyse respirometry data from AquaResp . (R package, Version 0.0.2) 10.5281/zenodo.20370947

[jfb70678-bib-0062] Wood, C. M. (2019). Internal spatial and temporal CO_2_ dynamics: Fasting, feeding, drinking, and the alkaline tide. In Fish physiology (Vol. 37, pp. 245–286). Academic Press.

[jfb70678-bib-0063] Wood, C. M. , Bucking, C. , Fitzpatrick, J. , & Nadella, S. (2007). The alkaline tide goes out and the nitrogen stays in after feeding in the dogfish shark, *Squalus acanthias* . Respiratory Physiology & Neurobiology, 159, 163–170. 10.1016/j.resp.2007.06.008 17656159

[jfb70678-bib-0064] Wood, C. M. , Kajimura, M. , Mommsen, T. P. , & Walsh, P. J. (2005). Alkaline tide and nitrogen conservation after feeding in an elasmobranch (*Squalus acanthias*). Journal of Experimental Biology, 208(14), 2693–2705. 10.1242/jeb.01678 16000539

[jfb70678-bib-0065] Yúfera, M. , & Darías, M. J. (2007). Changes in the gastrointestinal pH from larvae to adult in Senegal sole (*Solea senegalensis*). Aquaculture, 267(1–4), 94–99. 10.1016/j.aquaculture.2007.02.009

[jfb70678-bib-0066] Zahl, I. H. , Samuelsen, O. , & Kiessling, A. (2012). Anaesthesia of farmed fish: Implications for welfare. Fish Physiology and Biochemistry, 38, 201–218. 10.1007/s10695-011-9565-1 22160749

