# Supplementary material for: Method of meal delivery influences estimates of postprandial metabolism and alkaline tide in a benthic fish
Source: J Fish Biol. 2026 Sep 30;109(5):3003–18. doi: 10.1111/jfb.70678 (PMC13770699; doi:10.1111/jfb.70678)
Supplement: Supplementary file 1 [file JFB-109-3003-s001.pdf]

Supplementary Information

Method of meal delivery influences estimates of postprandial metabolism and alkaline tide in a benthic fish

Junlin Chen[1], Barrett W. Wolfe[1], Harriet R. Goodrich[1*]

[1]Institute for Marine and Antarctic Studies (IMAS), University of Tasmania, Hobart, TAS 7001, Australia

*Corresponding author: harriet.goodrich@utas.edu.au

Contents

Table S1. Summary of studies measuring postprandial fish physiology by taxon and meal delivery (voluntary feeding or intragastric gavage). Counts in each cell are numbers of studies using each method for each species, followed by superscript reference numbers corresponding to reference numbers in Table S2.

Table S2. Literature survey of primary research on fish postprandial physiology (1972–2025), classified by method of meal delivery. Studies were identified through Google Scholar and reference-list searches, and were included if they experimentally fed fish, measured a postprandial metabolic, acid-base, or digestive variable, and explicitly stated the feeding method. For each study, the table reports the species or taxonomic group, the method of meal delivery (voluntary feeding or intragastric gavage), the year of publication, and the full reference. See Materials and Methods for full inclusion and exclusion criteria.

Figure S1. Time series of oxygen consumption rate (MO_2) in sand flathead *(P. bassensis)* following feeding. Individual MO_2 time courses across batches (Batch 1–5), with each line representing one individual fish; colours denote treatment group (Fasted, dark grey; sham gavage, light grey; voluntary, blue; gavage, brown).

Table S1. Summary of studies measuring postprandial physiology by taxon and meal delivery (voluntary feeding or intragastric gavage). Counts in each cell are numbers of studies using each method for each species, followed by superscript reference numbers corresponding to reference numbers in Table S2.

Species / taxonomic group	Voluntary	Gavage
Anguilla anguilla	1[45]	1[4]
Anguilla rostrata	1[37]	—
Brachydanio/Danio rerio	1[41]	—
Catostomus commersonii	—	1[47]
Channa argus (juv)	1[1]	—
Coryphaena hippurus	1[76]	—
Ctenopharyngodon idella	1[55]	—
Cyprinus carpio	1[29]	—
Dicentrarchus labrax	1[62]	2[13,27]
Gadus morhua	3[23,25,54]	3[3,24,26]
Gadus morhua (juv)	—	1[10]
Harpagifer antarcticus	1[78]	—
Hippoglossus hippoglossus	1[38]	—
Ictalurus punctatus	—	1[36]
Kuhlia sandvicensis	1[61]	—
Lates calcarifer	1[60]	—
Leptocottus armatus	1[65]	—
Micropterus salmoides	1[46]	3[14,15,16]
Notothenia neglecta / *Myoxocephalus scorpius* / *Cirrhitichys bleekeri*	1[6]	—
Oncorhynchus mykiss	6[31,39,42,43,71,72]	7[30,32,33,48,57,64,69]
Oncorhynchus tshawytscha	1[67]	2[68,70]
Oreochromis niloticus	1[59]	—
Pagothenia borchgrevinki	—	1[66]
Paracirrhites arcatus / *Lutjanus fulvus*	1[8]	—
Pelteobagrus vachelli	1[53]	—
Platichthys flesus	1[9]	—
Pleuronectes platessa	1[17]	—
Polyprion oxygeneios	3[73,74,75]	—
Pterois spp.	1[77]	—
Salmo salar	2[56,58]	—
Salmonids (mixed)	—	1[7]
Sander vitreus	—	1[35]
Scyliorhinus canicula	1[34]	—
Silurus asotus	1[21]	—
Silurus meridionalis	9[18,19,20,22,40,49,50,51,52]	—
Siniperca chuatsi + *Channa argus*	1[2]	—
Sparus aurata	1[28]	—
Squalus acanthias	—	1[63]
Thunnus maccoyii	2[11,44]	—
Thunnus orientalis	1[12]	—
9 freshwater fish spp.	1[5]	—
**Total** (Proportion of total)	**53** (67.9%)	**25** (32.1%)

Table S2. Literature survey of primary research studies on fish postprandial physiology (1972–2025), classified by method of meal delivery. Studies were identified through Google Scholar and reference-list searches, and were included if they experimentally fed fish, measured a postprandial metabolic, acid-base, or digestive variable, and explicitly stated the feeding method. For each study, the table reports the species or taxonomic group, the method of meal delivery (voluntary feeding or intragastric gavage), the year of publication, and the full reference. See Materials and Methods for full inclusion and exclusion criteria.

No.	Citation	Species / group	Method	Year	Reference
**1**	Wang et al. 2012	*Channa argus* (juv)	Voluntary	2012	*Wang, Q., Wang, W., Huang, Q., Zhang, Y., & Luo, Y. (2012). Effect of meal size on the specific dynamic action of the juvenile snakehead (Channa argus). Comparative Biochemistry and Physiology Part A: Molecular & Integrative Physiology, 161(4), 401-405.*
**2**	Liu et al. 2000	*Siniperca chuatsi* + *Channa argus*	Voluntary	2000	*Liu, J., Cui, Y., & Liu, J. (2000). Resting metabolism and heat increment of feeding in mandarin fish (Siniperca chuatsi) and Chinese snakehead (Channa argus). Comparative Biochemistry and Physiology Part A: Molecular & Integrative Physiology, 127(2), 131-138.*
**3**	Tirsgaard et al. 2015a	*Gadus morhua*	Gavage	2015	*Tirsgaard, B., Moran, D., & Steffensen, J. F. (2015). Prolonged SDA and reduced digestive efficiency under elevated CO2 may explain reduced growth in Atlantic cod (Gadus morhua). Aquatic Toxicology, 158, 171-180.*
**4**	Methling et al. 2013	*Anguilla anguilla*	Gavage	2013	*Methling, C., Pedersen, P. B., Steffensen, J. F., & Skov, P. V. (2013). Hypercapnia adversely affects postprandial metabolism in the European eel (Anguilla anguilla). Aquaculture, 416, 166-172.*
**5**	Pang et al. 2024	9 freshwater fish spp.	Voluntary	2024	*Pang, X., Yang, J., Xiang, S. A., Sun, H., & Fu, S. J. (2024). Interspecific differences in growth, digestion, and feeding metabolism among freshwater fishes with different food habits. Frontiers in Marine Science, 11, 1490406.*
**6**	Johnston & Battram 1993	*Notothenia neglecta / Myoxocephalus scorpius / Cirrhitichys bleekeri*	Voluntary	1993	*Johnston, I. A., & Battram, J. (1993). Feeding energetics and metabolism in demersal fish species from Antarctic, temperate and tropical environments. Marine biology, 115(1), 7-14.*
**7**	Smith et al. 1978	Salmonids (mixed)	Gavage	1978	*Smith, R. R., Rumsey, G. L., & Scott, M. L. (1978). Heat increment associated with dietary protein, fat, carbohydrate and complete diets in salmonids comparative energetic efficiency. The Journal of nutrition, 108(6), 1025-1032.*
**8**	Brijs et al. 2025	*Paracirrhites arcatus / Lutjanus fulvus*	Voluntary	2025	*Brijs, J., Moore, C., Schakmann, M., Souza, T., Grellman, K., Tran, L. L., ... & Johansen, J. L. (2025). Eat more, often: The capacity of piscivores to meet increased energy demands in warming oceans. Science of The Total Environment, 973, 179105.*
**9**	Taylor et al. 2007	*Platichthys flesus*	Voluntary	2007	*Taylor, J. R., Whittamore, J. M., Wilson, R. W., & Grosell, M. (2007). Postprandial acid–base balance and ion regulation in freshwater and seawater-acclimated European flounder, Platichthys flesus. Journal of Comparative Physiology B, 177(6), 597-608.*
**10**	Chabot et al. 2016	*Gadus morhua* (juv)	Gavage	2016	*Chabot, D., Koenker, R., & Farrell, A. P. (2016). The measurement of specific dynamic action in fishes. Journal of Fish Biology, 88(1), 152-172.*
**11**	Fitzgibbon et al. 2007	*Thunnus maccoyii*	Voluntary	2007	*Fitzgibbon, Q. P., Seymour, R. S., Ellis, D., & Buchanan, J. (2007). The energetic consequence of specific dynamic action in southern bluefin tuna Thunnus maccoyii. Journal of Experimental Biology, 210(2), 290-298.*
**12**	Clark et al. 2010	*Thunnus orientalis*	Voluntary	2010	*Clark, T. D., Brandt, W. T., Nogueira, J., Rodriguez, L. E., Price, M., Farwell, C. J., & Block, B. A. (2010). Postprandial metabolism of Pacific bluefin tuna (Thunnus orientalis). Journal of Experimental Biology, 213(14), 2379-2385.*
**13**	Altimiras et al. 2008	*Dicentrarchus labrax*	Gavage	2008	*Altimiras, J., Claireaux, G., Sandblom, E., Farrell, A. P., McKenzie, D. J., & Axelsson, M. (2008). Gastrointestinal blood flow and postprandial metabolism in swimming sea bass Dicentrarchus labrax. Physiological and Biochemical Zoology, 81(5), 663-672.*

No.	Citation	Species / group	Method	Year	Reference
**14**	Beamish 1974	*Micropterus salmoides*	Gavage	1974	*Beamish, F. W. H. (1974). Apparent specific dynamic action of largemouth bass, Micropterus salmoides. Journal of the Fisheries Board of Canada, 31(11), 1763-1769.*
**15**	Tandler & Beamish 1979	*Micropterus salmoides*	Gavage	1979	*Tandler, A., & Beamish, F. W. H. (1979). Mechanical and biochemical components of apparent specific dynamic action in largemouth bass, Micropterm salmoides Lacéepède. Journal of Fish Biology, 14(4), 343-350.*
**16**	Tandler & Beamish 1980	*Micropterus salmoides*	Gavage	1980	*Tandler, A., & Beamish, F. W. H. (1980). Specific dynamic action and diet in largemouth bass, Micropterus salmoides (Lacepede). The Journal of nutrition, 110(4), 750-764.*
**17**	Jobling & Davies 1980	*Pleuronectes platessa*	Voluntary	1980	*Jobling, M., & Davies, P. S. (1980). Effects of feeding on metabolic rate, and the Specific Dynamic Action in plaice, Pleuronectes platessa L. Journal of Fish Biology, 16(6), 629-638.*
**18**	Fu et al. 2005a	*Silurus meridionalis*	Voluntary	2005	*Fu, S. J., Xie, X. J., & Cao, Z. D. (2005). Effect of meal size on postprandial metabolic response in southern catfish (Silurus meridionalis). Comparative Biochemistry and Physiology Part A: Molecular & Integrative Physiology, 140(4), 445-451.*
**19**	Fu et al. 2005b	*Silurus meridionalis*	Voluntary	2005	*Fu, S. J., Xie, X. J., & Cao, Z. D. (2005). Effect of feeding level and feeding frequency on specific dynamic action in Silurus meridionalis. Journal of fish biology, 67(1), 171-181.*
**20**	Fu et al. 2005c	*Silurus meridionalis*	Voluntary	2005	*Fu, S. J., Xie, X. J., & Cao, Z. D. (2005). Effect of dietary composition on specific dynamic action in southern catfish Silurus meridionalis Chen. Aquaculture Research, 36(14), 1384-1390.*
**21**	Fu et al. 2006	*Silurus asotus*	Voluntary	2006	*Fu, S. J., Cao, Z. D., & Peng, J. L. (2006). Effect of meal size on postprandial metabolic response in Chinese catfish (Silurus asotus Linnaeus). Journal of Comparative Physiology B, 176(5), 489-495.*
**22**	Luo & Xie 2008	*Silurus meridionalis*	Voluntary	2008	*Luo, Y., & Xie, X. (2008). Effects of temperature on the specific dynamic action of the southern catfish, Silurus meridionalis. Comparative Biochemistry and Physiology Part A: Molecular & Integrative Physiology, 149(2), 150-156.*
**23**	Soofiani & Hawkins 1982	*Gadus morhua*	Voluntary	1982	*Soofiani, N. M., & Hawkins, A. D. (1982). Energetic costs at different levels of feeding in juvenile cod, Gadus morhua L. Journal of Fish Biology, 21(5), 577-592.*
**24**	Jordan & Steffensen 2007	*Gadus morhua*	Gavage	2007	*Jordan, A. D., & Steffensen, J. F. (2007). Effects of ration size and hypoxia on specific dynamic action in the cod. Physiological and Biochemical Zoology, 80(2), 178-185.*
**25**	Tirsgaard et al. 2015b	Gadus morhua	Voluntary	2015	Tirsgaard, B., Svendsen, J. C., & Steffensen, J. F. (2015). Effects of temperature on specific dynamic action in Atlantic cod Gadus morhua. Fish Physiology and Biochemistry, 41(1), 41-50.
**26**	Behrens et al. 2012	*Gadus morhua*	Gavage	2012	*Behrens, J. W., Axelsson, M., Neuenfeldt, S., & Seth, H. (2012). Effects of hypoxic exposure during feeding on SDA and postprandial cardiovascular physiology in the Atlantic cod, Gadus morhua.*
**27**	Dupont-Prinet et al. 2009	*Dicentrarchus labrax*	Gavage	2009	*Dupont-Prinet, A., Claireaux, G., & McKenzie, D. J. (2009). Effects of feeding and hypoxia on cardiac performance and gastrointestinal blood flow during critical speed swimming in the sea bass Dicentrarchus labrax. Comparative Biochemistry and Physiology Part A: Molecular & Integrative Physiology, 154(2), 233-240.*
**28**	Remen et al. 2015	*Sparus aurata*	Voluntary	2015	*Remen, M., Nederlof, M. A., Folkedal, O., Thorsheim, G., Sitjà-Bobadilla, A., Pérez-Sánchez, J., ... & Olsen, R. E. (2015). Effect of temperature on the metabolism, behaviour and oxygen requirements of Sparus aurata. Aquaculture Environment Interactions, 7(2), 115-123.*
**29**	Chakraborty et al. 1995	*Cyprinus carpio*	Voluntary	1995	*Chakraborty, S. C., Ross, L. G., & Ross, B. (1995). Energy budget and metabolism in common carp, Cyprinus carpio L., fed on different dietary protein levels and at different ration levels. Aquaculture Nutrition, 1(3), 179-187.*

No.	Citation	Species / group	Method	Year	Reference
**30**	Cooper & Wilson 2008	*Oncorhynchus mykiss*	Gavage	2008	*Cooper, C. A., & Wilson, R. W. (2008). Post-prandial alkaline tide in freshwater rainbow trout: effects of meal anticipation on recovery from acid–base and ion regulatory disturbances. Journal of Experimental Biology, 211(15), 2542-2550.*
**31**	Eliason et al. 2007	Oncorhynchus mykiss	Voluntary	2007	*Eliason, E. J., Higgs, D. A., & Farrell, A. P. (2007). Effect of isoenergetic diets with different protein and lipid content on the growth performance and heat increment of rainbow trout. Aquaculture, 272(1-4), 723-736.*
**32**	Eliason et al. 2008	*Oncorhynchus mykiss*	Gavage	2008	*Eliason, E. J., Higgs, D. A., & Farrell, A. P. (2008). Postprandial gastrointestinal blood flow, oxygen consumption and heart rate in rainbow trout (Oncorhynchus mykiss). Comparative Biochemistry and Physiology Part A: Molecular & Integrative Physiology, 149(4), 380-388.*
**33**	Eliason & Farrell 2014	*Oncorhynchus mykiss*	Gavage	2014	*Eliason, E. J., & Farrell, A. P. (2014). Effect of hypoxia on specific dynamic action and postprandial cardiovascular physiology in rainbow trout (Oncorhynchus mykiss). Comparative Biochemistry and Physiology Part A: Molecular & Integrative Physiology, 171, 44-50.*
**34**	Sims & Davies 1994	*Scyliorhinus canicula*	Voluntary	1994	*Sims, D. W., & Davies, S. J. (1994). Does specific dynamic action (SDA) regulate return of appetite in the lesser spotted dogfish, Scyliorhinus caniculcla?. Journal of Fish Biology, 45(2), 341-348.*
**35**	Bihun et al. 2024	*Sander vitreus*	Gavage	2024	*Bihun, C. J., Stewart, E. M., Lechner, E. R., Brownscombe, J. W., & Raby, G. D. (2024). Thermal performance curves for aerobic scope and specific dynamic action in a sexually dimorphic piscivore: implications for a warming climate. Journal of Experimental Biology, 227(14), jeb247207.*
**36**	Brown & Cameron 1991	*Ictalurus punctatus*	Gavage	1991	*Brown, C. R., & Cameron, J. N. (1991). The induction of specific dynamic action in channel catfish by infusion of essential amino acids. Physiological zoology, 64(1), 276-297.*
**37**	Lee Gallagher & Matthews 1987	*Anguilla rostrata*	Voluntary	1987	*Lee Gallagher, M., & Matthews, A. M. (1987). Oxygen consumption and ammonia excretion of the American eel Anguilla rostrata fed diets with varying protein energy ratios and protein levels. Journal of the World Aquaculture Society, 18(2), 107-112.*
**38**	Lygre et al. 2025	*Hippoglossus hippoglossus*	Voluntary	2025	*Lygre, E. F., Gomes, A. S., Hess-Erga, O. K., Norberg, B., Nilsson, J., Perrichon, P., & Rønnestad, I. (2025). Gastrointestinal evacuation and return of appetite in Atlantic halibut (Hippoglossus hippoglossus L.). Aquaculture, 599, 742147.*
**39**	Beamish et al. 1986	Oncorhynchus mykiss	Voluntary	1986	*Beamish, F. W. H., Hilton, J. W., Niimi, E., & Slinger, S. J. (1986). Dietary carbohydrate and growth, body composition and heat increment in rainbow trout (Salmo gairdneri). Fish physiology and biochemistry, 1(2), 85-91.*
**40**	Fu et al. 2007	*Silurus meridionalis*	Voluntary	2007	*Fu, S. J., Cao, Z. D., & Peng, J. L. (2007). Effect of feeding and fasting on excess post-exercise oxygen consumption in juvenile southern catfish (Silurus meridionalis Chen). Comparative Biochemistry and Physiology Part A: Molecular & Integrative Physiology, 146(3), 435-439.*
**41**	Lucas & Priede 1992	*Brachydanio/Danio rerio*	Voluntary	1992	*Lucas, M. C., & Priede, I. G. (1992). Utilization of metabolic scope in relation to feeding and activity by individual and grouped zebrafish, Brachydanio rerio (Hamilton-Buchanan). Journal of Fish Biology, 41(2), 175-190.*
**42**	Cho et al. 1976	*Oncorhynchus mykiss*	Voluntary	1976	*Cho, C. Y., Slinger, S. J., & Bayley, H. S. (1976). Influence of level and type of dietary protein, and of level of feeding on feed utilization by rainbow trout. The Journal of nutrition, 106(11), 1547-1556.*
**43**	Adams et al. 2022	*Oncorhynchus mykiss*	Voluntary	2022	*Adams, O. A., Zhang, Y., Gilbert, M. H., Lawrence, C. S., Snow, M., & Farrell, A. P. (2022). An unusually high upper thermal acclimation potential for rainbow trout. Conservation Physiology, 10(1), coab101.*
**44**	Fitzgibbon & Seymour 2009	*Thunnus maccoyii*	Voluntary	2009	*Fitzgibbon, Q. P., & Seymour, R. S. (2009). Postprandial metabolic increment of southern bluefin tuna Thunnus maccoyii ingesting high or low-lipid sardines Sardinops sagax. Journal of Fish Biology, 75(7), 1586-1600.*

No.	Citation	Species / group	Method	Year	Reference
**45**	Owen 2001	*Anguilla anguilla*	Voluntary	2001	*Owen, S. F. (2001). Meeting energy budgets by modulation of behaviour and physiology in the eel (Anguilla anguilla L.). Comparative Biochemistry and Physiology Part A: Molecular & Integrative Physiology, 128(3), 629-642.*
**46**	Tandler & Beamish 1981	Micropterus salmoides	Voluntary	1981	*Tandler, A., & Beamish, F. W. H. (1981). Apparent specific dynamic action (SDA), fish weight and level of caloric intake in largemouth bass, Micropterus salmoides Lacepede. Aquaculture, 23(1-4), 231-242.*
**47**	Beamish & Trippel 1990	*Catostomus commersonii*	Gavage	1990	*Beamish, F. W. H., & Trippel, E. A. (1990). Heat increment: a static or dynamic dimension in bioenergetic models?. Transactions of the American Fisheries Society, 119(4), 649-661.*
**48**	Kaczanowski & Beamish 1996	*Oncorhynchus mykiss*	Gavage	1996	*Kaczanowski, T. C., & Beamish, F. W. H. (1996). Dietary essential amino acids and heat increment in rainbow trout (Oncorhynchus mykiss). Fish Physiology and Biochemistry, 15(2), 105-120.*
**49**	Fu et al. 2005d	*Silurus meridionalis*	Voluntary	2005	*Fu, S. J., Xie, X. J., & Cao, Z. D. (2005). Effect of fasting on resting metabolic rate and postprandial metabolic response in Silurus meridionalis. Journal of Fish Biology, 67(1), 279-285.*
**50**	Luo & Xie 2009a	*Silurus meridionalis*	Voluntary	2009	*Luo, Y., & Xie, X. (2009). Effects of body lipid content on the resting metabolic rate and postprandial metabolic response in the southern catfish Silurus meridionalis. Comparative Biochemistry and Physiology Part A: Molecular & Integrative Physiology, 154(4), 547-550.*
**51**	Luo & Xie 2009b	*Silurus meridionalis*	Voluntary	2009	*Luo, Y., & Xie, X. (2009). The effect of temperature on post-feeding ammonia excretion and oxygen consumption in the southern catfish. Journal of Comparative Physiology B, 179(6), 681-689.*
**52**	Pang et al. 2022	*Silurus meridionalis*	Voluntary	2022	*Pang, X., Tan, G., Sun, H., Shi, H. Y., Su, S. Q., Li, Y., & Fu, S. J. (2022). Effect of feeding different diets on postprandial metabolic response, digestive capacity and growth performance in juvenile southern catfish (Silurus meridionalis). Aquaculture Reports, 27, 101413.*
**53**	Li et al. 2010	*Pelteobagrus vachelli*	Voluntary	2010	*Li, X. M., Cao, Z. D., Peng, J. L., & Fu, S. J. (2010). The effect of exercise training on the metabolic interaction between digestion and locomotion in juvenile darkbarbel catfish (Peltebagrus vachelli). Comparative Biochemistry and Physiology Part A: Molecular & Integrative Physiology, 156(1), 67-73.*
**54**	Soofiani & Priede 1985	*Gadus morhua*	Voluntary	1985	*Soofiani, N. M., & Priede, I. G. (1985). Aerobic metabolic scope and swimming performance in juvenile cod, Gadus morhua L. Journal of Fish Biology, 26(2), 127-138.*
**55**	Carter & Brafield 1992	*Ctenopharyngodon idella*	Voluntary	1992	*Carter, C. G., & Brafield, A. E. (1992). The relationship between specific dynamic action and growth in grass carp, Ctenophavyngodon idella (Val.). Journal of Fish Biology, 40(6), 895-907.*
**56**	Millidine et al. 2009	*Salmo salar*	Voluntary	2009	*Millidine, K. J., Armstrong, J. D., & Metcalfe, N. B. (2009). Juvenile salmon with high standard metabolic rates have higher energy costs but can process meals faster. Proceedings of the Royal Society B: Biological Sciences, 276(1664), 2103-2108.*
**57**	Seth & Axelsson 2010	*Oncorhynchus mykiss*	Gavage	2010	*Seth, H., & Axelsson, M. (2010). Sympathetic, parasympathetic and enteric regulation of the gastrointestinal vasculature in rainbow trout (Oncorhynchus mykiss) under normal and postprandial conditions. Journal of Experimental Biology, 213(18), 3118-3126.*
**58**	Nuic et al. 2024	Salmo salar	Voluntary	2024	*Nuic, B., Bowden, A., Rombenso, A., Salini, M., Jago, M. K., Smullen, R., ... & Cramp, R. L. (2024). Effects of glutamine supplementation on Atlantic salmon Salmo salar metabolic performance at high temperatures. Aquaculture nutrition, 2024(1), 6632942.*
**59**	Moffatt et al. 2022	*Oreochromis niloticus*	Voluntary	2022	*Moffatt, K., Rossi, M., Park, E., Svendsen, J. C., & Wilson, J. M. (2022). Inhibition of gastric acid secretion with omeprazole affects fish specific dynamic action and growth rate: Implications for the development of phenotypic stomach loss. Frontiers in Physiology, 13, 966447.*

No.	Citation	Species / group	Method	Year	Reference
**60**	Goodrich et al. 2022a	*Lates calcarifer*	Voluntary	2022	*Goodrich, H. R., Wilson, R. W., Smullen, R., Barnes, A. C., & Franklin, C. E. (2022). Acidified fish feeds reduce the energetic and physiological costs of digestion in juvenile barramundi (Lates calcarifer). Aquaculture, 546, 737400.*
**61**	Muir & Niimi 1972	*Kuhlia sandvicensis*	Voluntary	1972	*Muir, B. S., & Niimi, A. J. (1972). Oxygen consumption of the euryhaline fish aholehole (Kuhlia sandvicensis) with reference to salinity, swimming, and food consumption. Journal of the Fisheries Board of Canada, 29(1), 67-77.*
**62**	Jourdan-Pineau et al. 2010	*Dicentrarchus labrax*	Voluntary	2010	*Jourdan-Pineau, H., Dupont-Prinet, A., Claireaux, G., & McKenzie, D. J. (2010). An investigation of metabolic prioritization in the European sea bass, Dicentrarchus labrax. Physiological and Biochemical Zoology, 83(1), 68-77.*
**63**	Wood et al. 2005	*Squalus acanthias*	Gavage	2005	*Wood, C. M., Kajimura, M., Mommsen, T. P., & Walsh, P. J. (2005). Alkaline tide and nitrogen conservation after feeding in an elasmobranch (Squalus acanthias). Journal of Experimental Biology, 208(14), 2693-2705.*
**64**	Rolland et al. 2015	*Oncorhynchus mykiss*	Gavage	2015	*Rolland, M., Larsen, B. K., Holm, J., Dalsgaard, J., & Skov, P. V. (2015). Effect of plant proteins and crystalline amino acid supplementation on postprandial plasma amino acid profiles and metabolic response in rainbow trout (Oncorhynchus mykiss). Aquaculture international, 23(4), 1071-1087.*
**65**	Wood et al. 2023	*Leptocottus armatus*	Voluntary	2023	*Wood, C. M., Wang, J., Jung, E. H., & Pelster, B. (2023). The physiological consequences of a very large natural meal in a voracious marine fish, the staghorn sculpin (Leptocottus armatus). Journal of Experimental Biology, 226(19), jeb246034.*
**66**	Sandblom et al. 2012	*Pagothenia borchgrevinki*	Gavage	2012	*Sandblom, E., Davison, W., & Axelsson, M. (2012). Cold physiology: postprandial blood flow dynamics and metabolism in the Antarctic fish Pagothenia borchgrevinki. PLoS One, 7(3), e33487.*
**67**	Thorarensen & Farrell 2006	*Oncorhynchus tshawytscha*	Voluntary	2006	*Thorarensen, H., & Farrell, A. P. (2006). Postprandial intestinal blood flow, metabolic rates, and exercise in Chinook salmon (Oncorhynchus tshawytscha). Physiological and Biochemical Zoology, 79(4), 688-694.*
**68**	Elvy et al. 2023	*Oncorhynchus tshawytscha*	Gavage	2023	*Elvy, J. E., Symonds, J. E., Hilton, Z., Walker, S. P., Tremblay, L. A., & Herbert, N. A. (2023). The relationships between specific dynamic action, nutrient retention and feed conversion ratio in farmed freshwater Chinook salmon (Oncorhynchus tshawytscha). Journal of Fish Biology, 102(3), 605-618.*
**69**	Bucking et al. 2009	*Oncorhynchus mykiss*	Gavage	2009	*Bucking, C., Fitzpatrick, J. L., Nadella, S. R., & Wood, C. M. (2009). Post-prandial metabolic alkalosis in the seawater-acclimated trout: the alkaline tide comes in. Journal of Experimental Biology, 212(14), 2159-2166.*
**70**	Lo et al. 2022	*Oncorhynchus tshawytscha*	Gavage	2022	*Lo, V. K., Martin, B. T., Danner, E. M., Cocherell, D. E., Cech, Jr, J. J., & Fangue, N. A. (2022). The effect of temperature on specific dynamic action of juvenile fall-run Chinook salmon, Oncorhynchus tshawytscha. Conservation physiology, 10(1), coac067.*
**71**	Segler et al. 2023	*Oncorhynchus mykiss*	Voluntary	2023	*Segler, P., Vanselow, K. H., Schlachter, M., Hasler, M., & Schulz, C. (2023). SDA coefficient is temperature dependent in rainbow trout (Oncorhynchus mykiss, Walbaum 1792) in a practical approach using group respirometry. Comparative Biochemistry and Physiology Part B: Biochemistry and Molecular Biology, 265, 110832.*
**72**	Goodrich et al. 2022b	*Oncorhynchus mykiss*	Voluntary	2022	*Goodrich, H. R., Berry, A. A., Montgomery, D. W., Davison, W. G., & Wilson, R. W. (2022). Fish feeds supplemented with calcium-based buffering minerals decrease stomach acidity, increase the blood alkaline tide and cost more to digest. Scientific Reports, 12(1), 18468.*
**73**	Khan et al. 2015	*Polyprion oxygeneios*	Voluntary	2015	*Khan, J. R., Pether, S., Bruce, M., Walker, S. P., & Herbert, N. A. (2015). The effect of temperature and ration size on specific dynamic action and production performance in juvenile hapuku (Polyprion oxygeneios). Aquaculture, 437, 67-74.*
**74**	Tromp et al. 2016	*Polyprion oxygeneios*	Voluntary	2016	*Tromp, J. J., Jones, P. L., Symonds, J. E., Walker, S. P., Pope, A., Pether, S. M., & Afonso, L. O. (2016). Effects of commercial diets and temperature on the growth performance and stress response of hapuku (Polyprion oxygeneios). Aquaculture, 452, 128-133.*

No.	Citation	Species / group	Method	Year	Reference
**75**	Khan et al. 2014	*Polyprion oxygeneios*	Voluntary	2014	*Khan, J. R., Pether, S., Bruce, M., Walker, S. P., & Herbert, N. A. (2014). Optimum temperatures for growth and feed conversion in cultured hapuku (Polyprion oxygeneios)—is there a link to aerobic metabolic scope and final temperature preference?. Aquaculture, 430, 107-113.*
**76**	Stieglitz et al. 2018	*Coryphaena hippurus*	Voluntary	2018	*Stieglitz, J. D., Benetti, D. D., & Grosell, M. (2018). Nutritional physiology of mahi-mahi (Coryphaena hippurus): Postprandial metabolic response to different diets and metabolic impacts on swim performance. Comparative Biochemistry and Physiology Part A: Molecular & Integrative Physiology, 215, 28-34.*
**77**	Steell et al. 2019	*Pterois* spp.	Voluntary	2019	*Steell, S. C., Van Leeuwen, T. E., Brownscombe, J. W., Cooke, S. J., & Eliason, E. J. (2019). An appetite for invasion: digestive physiology, thermal performance and food intake in lionfish (Pterois spp.). Journal of Experimental Biology, 222(19), jeb209437.*
**78**	Boyce & Clarke 1997	*Harpagifer antarcticus*	Voluntary	1997	*Boyce, S. J., & Clarke, A. (1997). Effect of body size and ration on specific dynamic action in the Antarctic plunderfish, Harpagifer antarcticus Nybelin 1947. Physiological Zoology, 70(6), 679-690.*

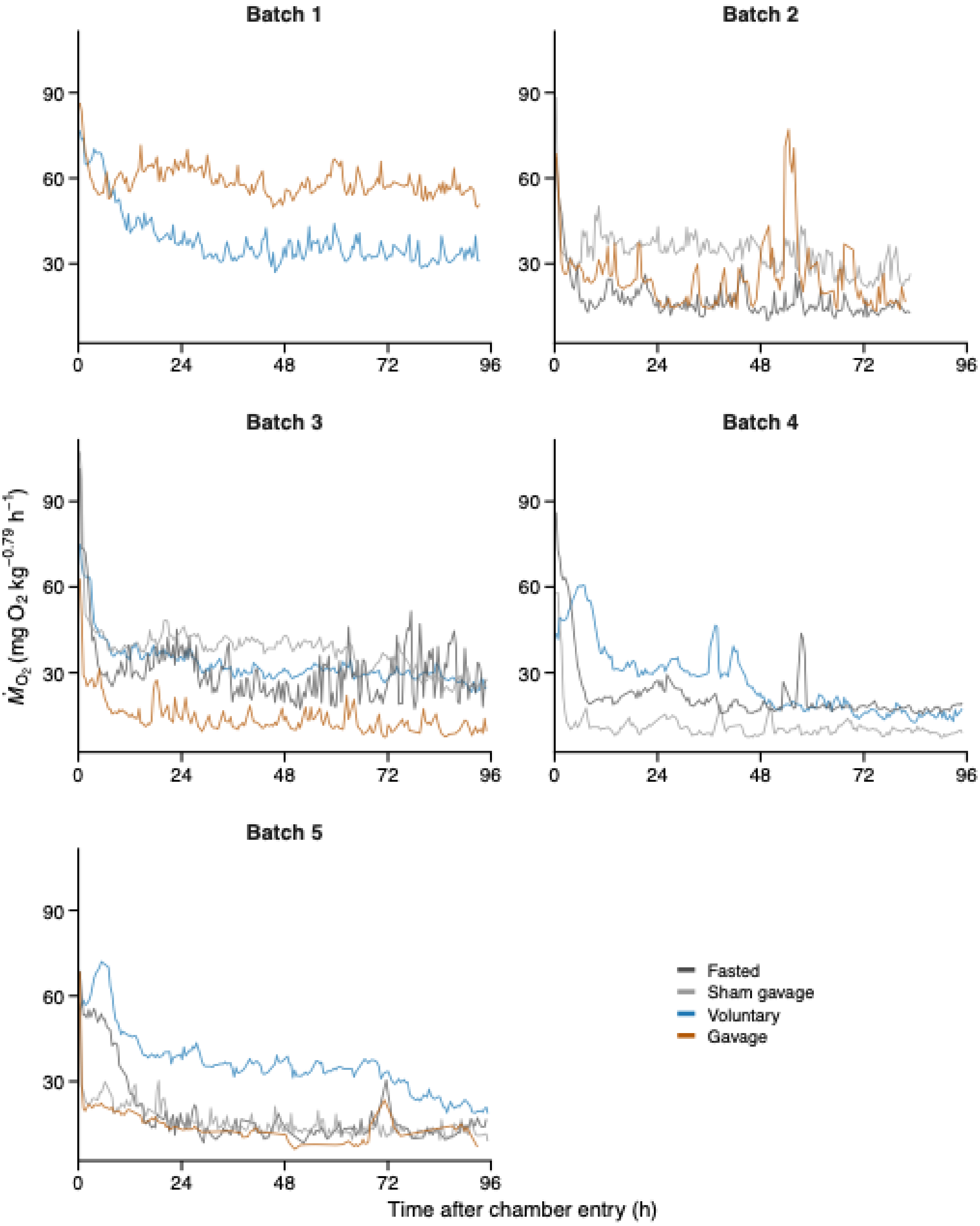

Figure S1. Time series of oxygen consumption rate (MO_2) in sand flathead *(P. bassensis)* following feeding. Individual MO_2 time courses across batches (Batch 1–5), with each line representing one individual fish; colours denote treatment group (Fasted, dark grey; Sham Gavage, light grey; Voluntary, blue; Gavage, brown).
